# Abstracts from Hydrocephalus 2016

**DOI:** 10.1186/s12987-017-0054-5

**Published:** 2017-06-07

**Authors:** A. Adam, J. Robison, J. Lu, R. Jose, N. Badran, T. Vivas-Buitrago, D. Rigamonti, A. Sattar, O. Omoush, M. Hammad, M. Dawood, M. Maghaslah, T. Belcher, K. Carson, J. Hoffberger, I. Jusué Torres, S. Foley, S. Yasar, Q. A. Thai, J. Wemmer, P. Klinge, L. Al-Mutawa, H. Al-Ghamdi, K. A. Carson, M. Asgari, D. de Zélicourt, V. Kurtcuoglu, S. Garnotel, S. Salmon, O. Balédent, A. Lokossou, G. Page, L. Balardy, Z. Czosnyka, P. Payoux, E. A. Schmidt, M. Zitoun, M. A. Sevestre, N. Alperin, I. Baudracco, C. Craven, S. Matloob, S. Thompson, P. Haylock Vize, L. Thorne, L. D. Watkins, A. K. Toma, Karl Bechter, A. C. Pong, L. Jugé, L. E. Bilston, S. Cheng, W. Bradley, F. Hakim, J. F. Ramón, M. F. Cárdenas, J. S. Davidson, C. García, D. González, S. Bermúdez, N. Useche, J. A. Mejía, P. Mayorga, F. Cruz, C. Martinez, M. C. Matiz, M. Vallejo, K. Ghotme, H. A. Soto, D. Riveros, A. Buitrago, M. Mora, L. Murcia, S. Bermudez, D. Cohen, D. Dasgupta, C. Curtis, L. Domínguez, A. J. Remolina, M. A. Grijalba, K. J. Whitehouse, R. J. Edwards, A. Eleftheriou, F. Lundin, K. N. Fountas, E. Z. Kapsalaki, H. F. Smisson, J. S. Robinson, M. J. Fritsch, W. Arouk, M. Garzon, M. Kang, K. Sandhu, D. Baghawatti, K. Aquilina, G. James, D. Thompson, M. Gehlen, M. Schmid Daners, A. Eklund, J. Malm, D. Gomez, M. Guerra, M. Jara, M. Flores, K. Vío, I. Moreno, S. Rodríguez, E. Ortega, E. M. Rodríguez, J. P. McAllister, M. M. Guerra, D. M. Morales, D. Sival, A. Jimenez, D. D. Limbrick, M. Ishikawa, S. Yamada, K. Yamamoto, A. Junkkari, A. Häyrinen, T. Rauramaa, H. Sintonen, O. Nerg, A. M. Koivisto, R. P. Roine, H. Viinamäki, H. Soininen, A. Luikku, J. E. Jääskeläinen, V. Leinonen, U. Kehler, O. Lilja-Lund, K. Kockum, E. M. Larsson, K. Riklund, L. Söderström, P. Hellström, K. Laurell, M. Kojoukhova, A. Sutela, R. Vanninen, K. I. Vanha, M. Timonen, J. Rummukainen, V. Korhonen, S. Helisalmi, E. Solje, A. M. Remes, J. Huovinen, J. Paananen, M. Hiltunen, M. Kurki, B. Martin, F. Loth, M. Luciano, A. J. Luikku, A. Hall, S. K. Herukka, J. Mattila, J. Lötjönen, I. Alafuzoff, I. Jurjević, M. Miyajima, M. Nakajima, H. Murai, T. Shin, D. Kawaguchi, C. Akiba, I. Ogino, K. Karagiozov, H Arai, R. C. Reis, M. J. Teixeira, C. G. Valêncio, D. da Vigua, L. Almeida-Lopes, M. W. Mancini, F. C. G. Pinto, R. H. Maykot, G. Calia, J. Tornai, S. S. S. Silvestre, G. Mendes, V. Sousa, B. Bezerra, P. Dutra, P. Modesto, M. F. Oliveira, C. E. Petitto, H. Pulhorn, A. Chandran, C. McMahon, A. S. Rao, M. Jumaly, D. Solomon, A. Moghekar, N. Relkin, M. Hamilton, H. Katzen, M. Williams, T. Bach, S. Zuspan, R. Holubkov, A. Rigamonti, G. Clemens, P. Sharkey, A. Sanyal, E. Sankey, K. Rigamonti, S. Naqvi, A. Hung, E. Schmidt, F. Ory-Magne, P. Gantet, A. Guenego, A. C. Januel, P. Tall, N. Fabre, L. Mahieu, C. Cognard, L. Gray, J. A. Buttner-Ennever, K. Takagi, K Onouchi, S. D. Thompson, L. D. Thorne, H. M. Tully, T. L. Wenger, W. A. Kukull, D. Doherty, W. B. Dobyns, D. Moran, S. Vakili, M. A. Patel, B. Elder, C. R. Goodwin, J. A. Crawford, M. V. Pletnikov, J. Xu, A. Blitz, D. A. Herzka, H. Guerrero-Cazares, A. Quiñones-Hinojosa, S. Mori, P. Saavedra, H. Treviño, K. Maitani, W. C. Ziai, V. Eslami, S. Nekoovaght-Tak, R. Dlugash, G. Yenokyan, N. McBee, D. F. Hanley

**Affiliations:** 1 Johns Hopkins University, Baltimore, MD USA; 2 Johns Hopkins Aramco Healthcare, Dhahran, Saudi Arabia; 3 Johns Hopkins Aramco Healthcare, Ras Tanura, Saudi Arabia; 4 Department of Neurosurgery, Johns Hopkins University, Baltimore, MD USA; 5 Department of Neurosurgery, Rhode Island Hospital, Providence, RI USA; 6 Department of Epidemiology, Johns Hopkins Bloomberg School of Public Health, Baltimore, MD USA; 7 The Interface Group, Institute of PhysiologyUniversity of Zurich, Zurich, Switzerland; 8 BioFlowImage Laboratory, University of Picardie Jules Verne, Amiens, France; 9 Reims Mathematics Laboratory, University of Reims Champagne-Ardenne, Reims, France; 10 Image Processing Laboratory, University Hospital of Amiens-Picardie, Amiens, France; 11 BioFlowImage Laboratory, Department of Medical Image Processing, University Hospital of Picardie Jules Verne, Amiens, France; 12 Department of Geriatric, University Hospital of Toulouse, Toulouse, France; 13 Neurosciences department, University of Cambridge, Cambridge, UK; 14 Department of Nuclear Medicine, University Hospital of Toulouse, Toulouse, France; 15 UMR 1214–INSERM/UPS–TONIC Toulouse Neuro-Imaging Center, Toulouse, France; 16 Department of Neurosurgery, University Hospital of Toulouse, Toulouse, France; 17 BioFlowImage, University Hospital of Picardie Jules Verne, Amiens, France; 18 University of Miami Health System, Miami, FL USA; 19 Victor Horsley Department of Neurosurgery, National Hospital for Neurology and Neurosurgery, London, UK; 20 Department Psychiatry II/Bezirkskliniken, Ulm University, Günzburg, Germany; 21 Neuroscience Research Australia, Randwick, Australia; 22 School of Medical Sciences, University of New South Wales, Kensington, Australia; 23 Prince of Wales Clinical School, University of New South Wales, Kensington, Australia; 24 Department of Engineering, Faculty of Science and Engineering, Macquarie University, Sydney, Australia; 25 Department of Radiology, University of California San Diego Health System, San Diego, CA USA; 26 Department of Surgery, Section of Neurosurgery, Fundación Santa Fe de Bogotá, Bogotá, Colombia; 27 Department of Diagnostic Imaging, Section of Neuroradiology, Fundación Santa Fe de Bogotá, Bogotá, Colombia; 28 Grupo de Hidrocefalia con Presión Normal, Hospital Universitario Fundación Santa Fe de Bogotá, Bogotá, Colombia; 29 Department of Microbiology, University College London Hospital NHS Foundation Trust, London, UK; 30 Neurosurgery Department, Cartagena University, Cartagena de Indias, Colombia; 31 Department of Paediatric Neurosurgery, Bristol Royal Hospital for Children, Bristol, UK; 32 Department of Neurology, University Hospital, Linköping, Sweden; 33 Division of Neuroscience, Department of Clinical and Experimental Medicine (IKE), Linköping University, Linköping, Sweden; 34 Department of Neurosurgery, School of Medicine, University of Thessaly, Larisa, Greece; 35 Department of Diagnostic Radiology, School of Medicine, University of Thessaly, Larisa, Greece; 36 Department of Neurosurgery, Georgia Neurosurgical Institute, Macon, GA USA; 37 Klinik für Neurochirurgie, Dietrich-Bonhoeffer-Klinikum, Neubrandenburg, Germany; 38 Great Ormond Street Hospital, London, UK; 39 Department of Mechanical and Process Engineering, ETH Zurich, Zurich, Switzerland; 40 Institute of Physiology, University of Zurich, Zurich, Switzerland; 41 Neuroscience Center Zurich and the Zurich Center for Integrative Human Physiology, University of Zurich, Zurich, Switzerland; 42 Department of Radiation Sciences, Umeå University, Umeå, Sweden; 43 Department of Pharmacology and Clinical Neuroscience, Umeå University, Umeå, Sweden; 44 Neurosurgery Department, Hospital Universitario, Fundación Santafe de Bogota, Bogota, Colombia; 45 Instituto de Anatomía, Histología y Patología, Facultad de Medicina, UACh, Valdivia, Chile; 46 Laboratorio de Polímeros, Facultad de Ciencias, UACh, Valdivia, Chile; 47 Instituto de Neurociencias Clínicas, Facultad de Medicina, UACh, Valdivia, Chile; 48 Department of Neurosurgery, St. Louis Children’s Hospital, St. Louis, MO USA; 49 Department of Pediatrics, St. Louis Children’s Hospital, St. Louis, MO USA; 50 Instituto de Histologia y Patologia, Facultad de Medicina, Universidad Austral de Chile, Valdivia, Chile; 51 Department of Pediatrics Pathology and Medical Biology, University Medical Center Groningen, University of Groningen, Groningen, The Netherlands; 52 Departamento de Biología Celular, Genética y Fisiología Facultad de Ciencias, Universidad de Malaga, Malaga, Spain; 53 Rakuwa Villa Ilios, Kyoto, Japan; 54 Normal Pressure Hydrocephalus Center, Otowa Hospital, Kyoto, Japan; 55 Department of Neurosurgery, Otowa Hospital, Kyoto, Japan; 56 Neurosurgery of NeuroCenter, Kuopio University Hospital and University of Eastern Finland, Kuopio, Finland; 57 Department of Pathology, Kuopio University Hospital and University of Eastern Finland, Kuopio, Finland; 58 Department of Public Health, University of Helsinki, Helsinki, Finland; 59 Neurology of NeuroCenter, University of Eastern Finland and Kuopio University Hospital, Kuopio, Finland; 60 University of Eastern Finland, Kuopio Finland and Helsinki and Uusimaa Hospital DistrictGroup Administration, Helsinki, Finland; 61 Department of Psychiatry, Kuopio University Hospital and University of Eastern Finland, Kuopio, Finland; 62 Department of Neurology, University of Eastern Finland, Kuopio, Finland; 63 Neurosurgical Department, Asklepios Klinik Hamburg Altona, Hamburg, Germany; 64 Department of Pharmacology and Clinical Neuroscience, Unit of Neurology, Östersund, Umeå University, Umeå, Sweden; 65 Department of Radiology, Uppsala University, Uppsala, Sweden; 66 Hydrocephalus Research Unit, Institute of Neuroscience and Physiology, The Sahlgrenska Academy, University of Gothenburg, Gothenburg, Sweden; 67 Department of Radiology, Kuopio University Hospital and University of Eastern Finland, Kuopio, Finland; 68 Unit of Neurology, Institute of Clinical Medicine, University of Eastern Finland, Kuopio, Finland; 69 Neurology of NeuroCenter, Kuopio University Hospital, Kuopio, Finland; 70 Department of Pathology, Kuopio University Hospital, Kuopio, Finland; 71 Institute of Clinical Medicine-Pathology, University of Eastern Finland, Kuopio, Finland; 72 Department of Neurosurgery, University of Eastern Finland and Kuopio University Hospital, Kuopio, Finland; 73 Institute of Clinical Medicine-Neurology, University of Eastern Finland, Kuopio, Finland; 74 Department of Neurology, Kuopio University Hospital, Kuopio, Finland; 75 Department of Neurosurgery, Kuopio University Hospital, Institute of Clinical Medicine, University of Eastern Finland, Kuopio, Finland; 76 Institute of Biomedicine, University of Eastern Finland, Kuopio, Finland; 77 Analytical and Translational Genetics Unit, Department of Medicine, Massachusetts General Hospital, Boston, MA USA; 78 Program in Medical and Population Genetics, Broad Institute of MIT and Harvard, Cambridge, MA USA; 79 Stanley Center for Psychiatric Research, Broad Institute for Harvard and MIT, Cambridge, MA USA; 80 Departments of Geriatric, University Hospital of Toulouse, Toulouse, France; 81 Biological Engineering, University of Idaho, Moscow, ID USA; 82 Mechanical Engineering, University of Akron, Akron, Ohio USA; 83 Neurosurgery, Johns Hopkins University, Baltimore, MA USA; 84 Neurosurgery of NeuroCenter, Kuopio University Hospital, Kuopio, Finland; 85 Department of Radiology, Kuopio University Hospital, Kuopio, Finland; 86 VTT Technical Research Centre of Finland, Tampere, Finland; 87 Combinostics Ltd, Tampere, Finland; 88 Rudbeck Laboratory, Department of Immunology, Genetics and Pathology, Uppsala University, Uppsala, Sweden; 89 Department of Pathology and Cytology, Uppsala University Hospital, Uppsala, Sweden; 90 Department of Neurosurgery, Graduate School of Medicine, Juntendo University, Tokyo, Japan; 91 Department of Pharmacology and Department of Neurology, University of Zagreb School of Medicine, Zagreb, Croatia; 92 Department of Neurosurgery, Graduate School of Medicine, Chiba University, Chiba, Japan; 93 Department of Neurosurgery, Juntendo University School of Medicine, Tokyo, Japan; 94 Group of Cerebral Hydrodynamics, Division of Functional Neurosurgery, Institute of Psychiatry, Hospital das Clínicas, University of São Paulo, São Paulo, Brazil; 95 Núcleo de Pesquisa e Ensino de Fototerapia nas Ciências da Saúde (NUPEN), São Carlos, Brazil; 96 Department of Neurosurgery, The Walton Centre, Liverpool, UK; 97 Department of Neuroradiology, The Walton Centre, Liverpool, UK; 98 The Johns Hopkins Hospital, Baltimore, MD USA; 99 Department of Neurology, Weill Cornell Medical College, New York, NY USA; 100 Department of Neurosurgery, University of Calgary, Alberta, Canada; 101 Department of Neurology, University of Miami, Miami, FL USA; 102 Department of Neurosurgery, Washington University, Seattle, WA USA; 103 Utah Data Collection Center (DCC), University of Utah, Salt Lake City, UT USA; 104 Cornell University, Ithaca, NY USA; 105 Bloomberg School of Public Health, Johns Hopkins University, Baltimore, MD USA; 106 School of Business, Loyola University Maryland, Baltimore, MD USA; 107 Primary Care, Johns Hopkins Aramco Healthcare, Ras Tanura, Saudi Arabia; 108 Primary Care, Johns Hopkins Aramco Healthcare, Abqaiq, Saudi Arabia; 109 Department of Neurosurgery, University Hospital Toulouse, Toulouse, France; 110 Department of Neurology, University Hospital Toulouse, Toulouse, France; 111 Department of Geriatry, University Hospital Toulouse, Toulouse, France; 112 Department of Nuclear Medicine, University Hospital Toulouse, Toulouse, France; 113 INSER TONIC 1014, Toulouse Neuroimaging Center, Toulouse, France; 114 Department of Neuroradiology, University Hospital Toulouse, Toulouse, France; 115 Department of Ophtalmology, University Hospital Toulouse, Toulouse, France; 116 Brain Physics Lab, Academic Neurosurgery, University of Cambridge, Cambridge, UK; 117 Neurology, Johns Hopkins Hospital, Baltimore, MD USA; 118 Department of Physiology, Johns Hopkins University, School of Medicine, Baltimore, MD USA; 119 Anatomy and Cell Biology, Ludwig-Maximilians-University, Munich, Germany; 120 Department of Neurosurgery, Johns Hopkins Hospital, Baltimore, MD USA; 121 Department of Neurology, Johns Hopkins Bayview Medical Center, Baltimore, MD USA; 122 Normal Pressure Hydrocephalus Center, Kashiwa-Tanaka Hospital, Kashiwa, Japan; 123 Department of Neurology, Kashiwa-Tanaka Hospital, Kashiwa, Japan; 124 The National Hospital for Neurology and Neurosurgery, Queen Square, London, UK; 125 Department of Neurology, University of Washington, Seattle, WA USA; 126 Department of Pediatrics, University of Washington, Seattle, WA USA; 127 Department of Epidemiology, University of Washington, Seattle, WA USA; 128 Department of Neurosurgery, Johns Hopkins University, School of Medicine, Baltimore, MD USA; 129 Johns Hopkins Biostatistics Center, Johns Hopkins Bloomberg School of Public Health, Baltimore, MD USA; 130 Johns Hopkins Bloomberg School of Public Health, Baltimore, MD USA; 131 Department of Psychiatry and Behavioral Sciences, Johns Hopkins University, School of Medicine, Baltimore, MD USA; 132 F. M. Kirby Research Center for Functional Brain Imaging at the Kennedy Krieger Institute, Johns Hopkins University, School of Medicine, Baltimore, MD USA; 133 Department of Radiology and Radiological Science, Johns Hopkins University, School of Medicine, Baltimore, MD USA; 134 Department of Biomedical Engineering, Johns Hopkins University, Baltimore, MD USA; 135 Department of Oncology, Johns Hopkins University, School of Medicine, Baltimore, MD USA; 136 Department of Radiology-Magnetic Resonance Research, Johns Hopkins University, School of Medicine, Baltimore, MD USA; 137 Johns Hopkins Hopkins Aramco Healthcare, Dhahran, Saudi Arabia; 138 Tohoku University School of Medicine, Sendai, Japan; 139 Department of Neurology, The Johns Hopkins University School of Medicine, Baltimore, MD USA; 140 Department of Biostatistics, The Johns Hopkins University Bloomberg School of Public Health, Baltimore, MD USA

## A1 Barriers to standardizing care for hydrocephalus in the Middle East

### A. Adam^1^, J. Robison^1^, J. Lu^1^, R. Jose^2^, N. Badran^2^, T. Vivas-Buitrago^1^, D. Rigamonti^1^

#### ^1^Johns Hopkins University, Baltimore, MD, USA; ^2^Johns Hopkins Aramco Healthcare, Dhahran, Saudi Arabia

##### **Correspondence:** A. Adam

*Fluids and Barriers of the CNS* 2017, 14(Suppl 1):A1

**Introduction:** Among the confirmed cases of hydrocephalus in the Middle East, there is no standardization of care or differentiation between various types of hydrocephalus. This is the first study in the Middle Eastern regions that aims at developing a hydrocephalus clinical protocol for appropriate standard of care.

**Methods:** We used a well-established clinical protocol from Johns Hopkins University and adapted it to a locally relevant clinical context with the help of an interdisciplinary panel of experts. The final protocol included diagnostic radiology studies, lumbar punctures (LP) as indication for surgery in hydrocephalus patients, and standardized times for follow-up visits were scheduled for optimal management of care. Baseline and follow-up testing protocol included gait assessment scales [Tinetti Gait and Balance Scale, Timed Up and Go task (TUG)] and ICIQ Urinary Incontinence short form.

**Results:** Key highlights were cultural and language barriers which resulted in editing the testing and measures for use within the region (ex. translation into Arabic, religious beliefs, and education levels were incorporated to modify protocols). Gender roles and space constraints restricted feasibility of gait testing and required invasive procedures (ex. TUG times could not be assessed in a public hallway for women and women demanded inpatient stays for LP).

**Conclusions:** The study highlights the need to raise awareness and standardize clinical protocols for screening and treating NPH in the region. A critical analysis of issues related to the how and why of implementation, and in turn optimizing the appropriateness and relevance to the particular circumstances is essential.

## A2 Evaluating burden for of normal pressure hydrocephalus (nph) in the Middle East using a population screening method

### A. Adam^1^, J. Lu^1^, J. Robison^1^, A. Sattar^3^, O. Omoush^3^, M. Hammad^3^, M. Dawood^3^, M. Maghaslah^3^, T. Belcher^3^, R. Jose^2^, N. Badran^2^, T. Vivas-Buitrago^1^, D. Rigamonti^1^

#### ^1^Johns Hopkins University, Baltimore, MD, USA; ^2^Johns Hopkins Aramco Healthcare, Dhahran, Saudi Arabia; ^3^Johns Hopkins Aramco Healthcare, Ras Tanura, Saudi Arabia

##### **Correspondence:** A. Adam

*Fluids and Barriers of the CNS* 2017, 14(Suppl 1):A2

**Introduction:** The burden of the geriatric population within Saudi Arabia is increasing rapidly and there is poor understanding of the prevalence of normal pressure hydrocephalus (NPH). This is the first study in the Middle Eastern regions that aims at diagnosing and increasing awareness of NPH through a population based screening approach.

**Methods:** The team implemented a nurse-based screening clinic within primary care at four clinic sites over 6 months. Patients were screened using standard gait assessment measures. Patients with symptomology indicative of Hydrocephalus were then sent for CT and then MRI, if necessary.

**Results:** The team screened over 300 patients in the first 6 months, of which, 31 were referred for head CT to confirm ventriculomegaly and diagnosis of NPH. Patient reluctance was high given the association between their symptoms and the progression of natural aging. Infrastructure to support screening (ex. radiological services) was also limited.

**Conclusions:** While the main focus of this study was in understanding the burden of NPH in the region, key areas were identified to encourage proactive screening of patients amongst primary care to raise awareness and further educate the country about hydrocephalus. Education was expanded on both the provider level as well as to the patient and family. From these screening measures, the team was able to train nurses to integrate screening for hydrocephalus within primary care. The team also raised awareness to physicians to refer patients with the triad to the new hydrocephalus clinic.

## A3 Complications post ventricular-atrial and ventricular-peritoneal shunt in normal pressure hydrocephalus patients: results over a 2-year follow-up from a randomized control trial

### A. Adam^1^, J. Lu^1^, J. Robison^1^, K. Carson^1^, J. Hoffberger^1^, I. Jusue Torres^1^, T. Vivas Buitrago^1^, S. Foley^2^, S. Yasar^1^, Q. A. Thai^1^, J. Wemmer^1^, P. Klinge^1^, D. Rigamonti^1^

#### ^1^Department of Neurosurgery, Johns Hopkins University, Baltimore, MD, USA; ^2^Department of Neurosurgery, Rhode Island Hospital, Providence, RI, USA

##### **Correspondence:** A. Adam

*Fluids and Barriers of the CNS* 2017, 14(Suppl 1):A3

**Introduction:** Ventricular-atrial and ventricular-peritoneal shunts are commonly employed treatment of normal pressure hydrocephalus (NPH). Few studies have focused on the complications at varied time points post-shunting surgery. This study aims to determine (1) description of complications seen post-shunting procedures and (2) if complications are associated with worsening of gait or functional independence post-surgery.

**Methods:** Between 2013 and 2016, 31 patients were enrolled at Rhode Island Hospital or Johns Hopkins Medical Institution for shunt surgery. Patients were eligible for the randomized, double blinded study if they were candidates for shunt surgery based on CSF drainage and radiological findings. They were followed up intermittently over a 2 year period for primary outcomes: Tinetti Gait and Balance Scale and Timed Up and Go task (TUG), Clinical outcomes: European iNPH scale (E-iNPH), Kiefer Index and Kubo- INPH grading Scales and Functional independence using the Barthel Index (BI) and the Modified Rankin Scale (MRS).

**Results:** (i) In 2 years post shunting, 9 complications occurred amongst the 31 patients (ii) Subdural hematoma (SDH) was most common complication seen in 3 patients followed by 2 patients with headaches and hearing loss each (iii) 63.64% of these complications occurred within 6 weeks post shunting. TUG and Tinetti were significantly different (p value <0.01) in patients with SDH. Patients without SDH had significant improvements in TUG and Tinetti scores (p value <0.001) while patients with SDH did not show significant improvements over the 2-years.

**Conclusions:** SDH is the most common complication seen post-shunting surgery and shows reduced and even worsening of gait improvements in NPH patients post-surgery.

## A4 Establishing a geriatric registry in the Middle East to identify risk factors and manifestations of normal-pressure hydrocephalus

### A. Adam^1^, J. Lu^1^, J. Robison^1^, A. Sattar^3^, O. Omoush^3^, M. Hammad^3^, M. Dawood^3^, M. Maghaslah^3^, T. Belcher^3^, R. Jose^2^, N. Badran^2^, L. Al-Mutawa^4^, H. Al-Ghamdi^5^, T. Vivas-Buitrago^1^, D. Rigamonti^1^

#### ^1^Department of Neurosurgery, Johns Hopkins University, Baltimore, MD, USA; ^2^Department of Neurosurgery, Johns Hopkins Aramco Healthcare, Dhahran, Saudi Arabia; ^3^Department of Primary Care, Johns Hopkins Aramco Healthcare, Ras Tanura, Saudi Arabia; ^4^Department of Primary Care, Johns Hopkins Aramco Healthcare, Al Hasa, Saudi Arabia; ^5^Department of Primary Care, Johns Hopkins Aramco Healthcare, Abqaiq, Saudi Arabia

##### **Correspondence:** A. Adam

*Fluids and Barriers of the CNS* 2017, 14(Suppl 1):A4

**Introduction:** With rapid aging of the Saudi society, diagnosis of normal-pressure hydrocephalus (NPH) becomes relevant. Little is known about vascular risk factors in Saudi Arabia (KSA). This study looks at comorbidities identified through establishing a geriatric registry in the region.

**Methods:** We collected and recorded from the medical record system, demographics, medications, co-morbidities of every patient aged 65 or more. Then patients scheduled for a regular clinic visit were evaluated and entered into the Registry, after performing a baseline functional screen using Tinetti Gait and Balance Scale, Timed Up and Go task (TUG), ICIQ Urinary Incontinence short form, and Picture Memory Impairment Screen.

**Results:** The team screened 333 patients in the first 6 months. The mean age of the patient population was 74.3 years old and 55.1% were male. 31.1% of the patients reported either being a current smoker or having smoked in the past and 58.8% having never smoked. Looking at clinical risk factors and comorbidities, 83.2% of the patients were either overweight or obese. 68.5 and 67.9% population reported being diagnosed with diabetes or hypertension respectively. 50.2% of the population reported being diagnosed with hyperlipidemia as well. Coronary artery diseases (57.9%) was the most commonly reported in patients with either diabetes, hypertension or hyperglycemia.

**Conclusions:** The study showcases the importance of setting a registry to measure the burden of co-morbidities in the elderly of Saudi Arabia. Proactively measuring the baseline degree of functional independence offers an optimal screening tool for the early diagnosis of NPH.

## A5 Assessing within patient improvement in gait and clinical measures in response to active shunt compared to placebo (non-active shunt)

### A. Adam^1^, J. Lu^1^, J. Robison^1^, K. A. Carson^2^, J. Hoffberger^1^, I. Jusue Torres^1^, T. Vivas Buitrago^1^, S. Foley^3^, S. Yasar^1^, Q. A. Thai^1^, J. Wemmer^1^, P. Klinge^1^, D. Rigamonti^1^

#### ^1^Department of Neurosurgery, Johns Hopkins University, Baltimore, MD, USA; ^2^Department of Epidemiology, Johns Hopkins Bloomberg School of Public Health, Baltimore, MD, USA; ^3^Department of Neurosurgery, Rhode Island Hospital, Providence, RI, USA

##### **Correspondence:** A. Adam

*Fluids and Barriers of the CNS* 2017, 14(Suppl 1):A5

**Introduction:** Normal pressure hydrocephalus (NPH) is a treatable cause of dementia, gait apraxia and urinary incontinence that results in significant disability if not identified and treated. However, response rates to shunting have been reported to vary between 31 and 89%. Furthermore, long term follow-up studies have shown loss of response to shunt which may be due to the natural history of disease, progression of comorbidities or a short term placebo effect following shunt surgery. In this abstract we look to determine within patient improvement in response to active shunt compared to placebo (non-active shunt).

**Methods:** This study prospectively studies 32 eligible patients from shunt surgeries conducted between 2013 and 2016 and were evaluated pre-surgery and 6-months post-surgery. Primary outcomes were change in gait assessed by the Tinetti Gait and Balance Scale and Timed Up and Go task (TUG). Clinical outcomes were Kiefer Index and Kubo-INPH grading Scales. Linear fixed effects models were used for analysis using STATA 13.0.

**Results:** Patients’ median age was 72 years (range 64–83) and 63% were male. All measures showed significant statistical improvement (p value 0.001) between baseline (no-shunt) versus 6-month (shunt). The largest improvement in gait measures were seen on the (Estimation: 1.8, p value <0.001) and Kudo on the functional scale (Estimation: 1.3, p value <0.001). In adjusting for pre-surgical characteristics, age at surgical procedure and BMI status were significantly associated with all measures (p value <0.01) expect the Tinetti scale.

**Conclusions:** Active shunting shows significant improvements in gait and clinical outcomes measures in patients NPH.

## A6 Assessment of commonly used scales to evaluate normal pressure hydrocephalus (nph) post shunt surgery

### A. Adam^1^, J. Lu^1^, J. Robison^1^, K. A. Carson^2^, J. Hoffberger^1^, I. JusueTorres^1^, T. Vivas-Buitrago^1^, S. Foley^3^, S. Yasar^1^, Q. A. Thai^1^, J. Wemmer^1^, P. Klinge^1^, D. Rigamonti^1^

#### ^1^Department of Neurosurgery, Johns Hopkins University, Baltimore, MD, USA; ^2^Department of Epidemiology, Johns Hopkins Bloomberg School of Public Health, Baltimore, MD, USA; ^3^Department of Neurosurgery, Rhode Island Hospital, Providence, RI, USA

##### **Correspondence:** A. Adam

*Fluids and Barriers of the CNS* 2017, 14(Suppl 1):A6

**Introduction:** Gait improvement after shunt placement has been well documented in normal pressure hydrocephalus (NPH); however, there is no preferred test to evaluate the extent and pattern of postsurgical improvements. To lessen the clinician and patient burden and time, we aimed to determine the sensitivity of the European iNPH scale (E-iNPH) to capture multiple domains of clinical improvement in NPH patients.

**Methods:** 30 eligible patients with diagnosed NPH, underwent shunting procedures and were evaluated pre-shunt and 6-month post-shunt. Gait was assessed by the Tinetti Gait and Balance Scale and Timed Up and Go task (TUG). Clinical outcomes were assessed using E-iNPH, Kudo iNPH scale and the Kiefer Index. McNemar’s test and Pearson’s Correlation were used to assess sensitivity between tests.

**Results:** At 6 months post-shunting, 90 and 71% of patients showed improvements on the E-INPH and Keifer tests, respectively. 67 and 73% of patients, using the TUG and Tinetti respectively, showed gait specific improvements. Improvements on E-INPH correlated closely with other tests: Kudo (0.70; p < 0.001), Tinetti (0.67; p < 0.001), TUG (0.78; p < 0.001), and Kiefer (0.74; p < 0.001). Comparisons on the McNemar’s test, the E-INPH scale is more sensitive in identifying NPH improvements at 6 months than other tests: Tinetti (*X* ^2^ = 5; p = 0.02), TUG (*X* ^2^ = 7; p < 0.001), and Kiefer (*X* ^2^ = 4.5; p = 0.02).

**Conclusions:** The results of this prospective multicenter study on patients with NPH supports the use of E-INPH scale in assessing improvements post shunt surgery. The E-INPH scale is more sensitive than other clinical tests in identifying improvement in patients at 6-months post shunt surgery.

## A7 Does glymphatic solute transport need paravascular bulk flow?

### M. Asgari, D. de Zélicourt, V. Kurtcuoglu

#### The Interface Group, Institute of Physiology, University of Zurich, Zurich, Switzerland

##### **Correspondence:** M. Asgari

*Fluids and Barriers of the CNS* 2017, 14(Suppl 1):A7

**Introduction:** Experimental observations of fast transport of fluorescent tracers in the cerebral cortex have led to the hypothesis of bulk water flow directed from arterial to venous paravascular spaces (PVS) through the cortical interstitium. The corresponding overall transport pathway has been named ‘glymphatic system’. The hypothesized glymphatic pathway requires the presence of a net driving force between the arterial and venous PVS to enable bulk flow. While this force has not been identified so far, arterial pulsation has been suggested as its origin because unilateral ligation of the internal carotid artery has been shown to decrease the paravascular solute transport, presumably owing to reduced pulsation of cerebral arteries.

**Methods:** We designed two distinct computational models (1) to assess the impact of arterial pulsation on water and solute dynamics in the para-arterial space and (2) to explore solute transport from para-arterial to the para-venous spaces through the cortical interstitium.

**Results:** We show that arterial pulsation is an unlikely contributor to the hypothetical driving force for bulk flow in the PVS. However, we further show that such pulsation may still lead to fast para-arterial solute transport through dispersion and without directed bulk flow.

**Conclusions:** Rapid paravascular solute transport with rates faster than diffusion does not require bulk flow through the paravascular space. Instead, dispersion, which is the combined effect of diffusion and localized flow, may enable fast solute transport. Dispersion may reconcile a number of apparently conflicting experimental observations, notably bidirectional solute transport in the PVS, which are not explained by the glymphatic hypothesis.

## A8 Numerical modeling of the cerebrospinal system: MRI and simulation to determine hydrocephalus mechanisms

### S. Garnotel^1,2,3^, S. Salmon^2^, O. Balédent^1,3^

#### ^1^BioFlowImage Laboratory, University of Picardie Jules Verne, Amiens, France;^ 2^Reims Mathematics Laboratory, University of Reims Champagne-Ardenne, Reims, France;^ 3^Image Processing Laboratory, University Hospital of Amiens-Picardie, Amiens, France

##### **Correspondence:** O. Balédent

*Fluids and Barriers of the CNS* 2017, 14(Suppl 1):A8

**Introduction:** Intracranial pressure (ICP) is an important clinical parameter for the proper functioning of the brain but this pressure is still difficult to measure in a non-invasive way and its regulation is quite hard to understand. Phase-contrast MRI (PC-MRI) provides arterial, venous and cerebrospinal fluid (CSF) cerebral flows in a noninvasive way that quantifies most of the fluid exchanges in the cranium. Fluid–structure interactions are numerous in the cerebrospinal system and difficult to understand in the rigid skull, so this study focussed on a numerical model of the cerebrospinal system taking into account CSF compartment, blood compartment and brain in fluid–structure interaction using PC-MRI measurements to approximate ICP.

**Methods:** A fluid–structure interaction model was designed by coupling the fluid mechanics equations and the solid mechanics equations to obtain a global representation of the cerebrospinal system. The input of this model is the PC-MRI measured flows in arteries, in veins and in the CSF at the cervical level. This model allows quantification of velocity, pressure and displacement in the whole system. Different configurations of the model were tested to evaluate their impact on the system.

**Results:** Ventricle stroke volume is widely impacted by heart rate and mechanical properties of the brain (Youngs modulus). Ventricles volume is modified under specific model configurations during a relatively short time (few minutes).

**Conclusions:** The presented model allows a quantification of the system variables and highlights significant variations of the stroke volume in the ventricles under parameters variations that can be an origin of hydrocephalus.

## A9 Assessment of the dynamic of the cerebrospinal fluid in the spinal canal of hydrocephalus patients

### O. Balédent^1^, A. Lokossou^1^, S. Garnotel^1^, G. Page^1^, L. Balardy^2^, Z. Czosnyka^3^, P. Payoux^4^, E. A. Schmidt^5,6^

#### ^1^BioFlowImage Laboratory, Department of Medical Image Processing, University Hospital of Picardie Jules Verne, Amiens, France; ^2^Department of Geriatric, University Hospital of Toulouse, Toulouse, France; ^3^Neurosciences department, University of Cambridge, Cambridge, UK; ^4^Department of Nuclear Medicine, University Hospital of Toulouse, Toulouse, France; ^5^UMR 1214–INSERM/UPS–TONIC Toulouse Neuro-Imaging Center, Toulouse, France; ^6^Department of Neurosurgery, University Hospital of Toulouse, Toulouse, France

##### **Correspondence:** O. Balédent

*Fluids and Barriers of the CNS* 2017, 14(Suppl 1):A9

**Introduction:** During cardiac cycle (CC), the vascular brain expansion is quickly compensated by cerebrospinal fluid (CSF) volume flush, also called the stroke volume (SVcsf), toward the spinal compartment. Change in this flush resulting from the modification of biomechanical properties of the spinal canal could alter the main compliance of the system. We aimed to calculate the SVcsf.

**Methods:** 83 subjects (74 ± 7 years) suspected of hydrocephalus underwent an infusion test. The change in intracranial pressure (ICP) at rest over the CC (ΔP_rest) was assessed from ICP monitoring before infusion using ICM+. After infusion, resistance to CSF outflow (R_out_) was calculated and was used to classify the patients in normal or pathological group. The day before the infusion, at the cervical level, the patients underwent a phase contrast MRI for the quantification of the spinal CSF flow oscillations. Then, SV_CSF_ was calculated and represents the CSF volume moving outside and inside the cranium during CC.

**Results:** 32 patients with a normal R_out_ (9 ± 2 mmHg/ml/min) were included in the Normal_inf_group. 51 others whose R_out_ was elevated (23 ± 14 mmHg/ml/min) were included in the Patho_inf_group. SVcsf was higher (p = 0.04) in the Normal_inf_group (0.62 ± 0.22 ml) than in the Patho_inf_group (0.52 ± 0.19 ml). ΔP_rest was smaller (p = 0.04) in the Normal_inf_group (1.92 ± 1.03 mmHg) than in the Patho_inf_group (2.46 ± 1.34 mmHg).

**Conclusions:** We found that the increase of R_out_ is associated with a decrease of the SVcsf combined with an increase of ΔP_rest. This decrease of SVcsf could be explained by: a spinal canal compliance decrease, a vascular brain expansion decrease or an increase of subarachnoid space resistance to CSF flow.

## A10 Cerebral venous drainage dynamics: intracranial extracranial comparison in upright and supine

### O. Balédent^1^, M. Zitoun^1^, A. Lokossou^1^, M. A. Sevestre^1^, N. Alperin^2^

#### ^1^BioFlowImage, University Hospital of Picardie Jules Verne, Amiens, France; ^2^University of Miami Health System, Miami, FL, USA

##### **Correspondence:** O. Balédent

*Fluids and Barriers of the CNS* 2017, 14(Suppl 1):A10

**Introduction:** It is well documented that venous drainage modulates intracranial pressure. It is less known how changes in body posture affect the link between intracranial and extracranial venous dynamics.

This study combines velocity encoding MRI and Doppler ultrasound to characterize the relationship between intracranial and extracranial venous dynamics and postural influence on the venous drainage dynamics.

**Methods:** Ten volunteers underwent Phase Contrast MRI study to measure venous drainage through the sagittal sinus as well as the extracranial drainage through the internal jugular veins. Flow waveform dynamics and mean flow rates were assessed to determine pulsatility [(flow_max-flow_min)/mean_flow] differences between intra and extra cranial flows.

B-mode Ultrasound study were used to assess the change of the internal jugular veins in the cross sectional area and Doppler ultrasound was used to asses flow velocities waveforms and estimated the mean flow.

**Results:** Ultrasound-derived measurements demonstrated a significant reduction in the extracranial drainage through the internal jugular veins in the upright posture compared with supine posture (130 ±60 vs. 20 ± 30 ml/min for the left side and 290 ± 380 vs. 60 ± 100 ml/min).

Comparison between intracranial and extracranial venous flow dynamics demonstrated significantly larger magnitude of pulsatility in the internal jugular veins (0.41 ± 0.13) than in the sagittal sinus (0.27 ± 0.08). These differences were evident regardless of large inter-individual variability.

**Conclusions:** These results emphasize the complexity of the venous modulation of the intracranial physiology and a potential role for the right side of the heart and the jugular valves in the overall intracranial physiology and intracranial pressure dynamics.

## A11 MPH in NPH: how much does walking speed increase after VP shunt insertion?

### I. Baudracco, C. Craven, S. Matloob, S. Thompson, P. Haylock Vize, L. Thorne, L. D. Watkins, AK Toma

#### Victor Horsley Department of Neurosurgery, National Hospital for Neurology and Neurosurgery, London, UK

##### **Correspondence:** I. Baudracco

*Fluids and Barriers of the CNS* 2017, 14(Suppl 1):A11

**Introduction:** Improvement in mobility is key aim of cerebrospinal fluid (CSF) diversion in idiopathic normal pressure hydrocephalus (INPH). However, there is little information on the estimated walking speed improvement expected after the insertion of a ventriculoperitoneal shunt (VP). In this study we measured actual improvement in walking speed, in miles per hour (MPH), after VP shunt insertion.

**Methods:** Single centre analysis of patients with probable INPH. Pre-and post-operative (following ventriculoperitoneal shunt insertion) walking speeds over 10 m were recorded. The speed over 10 m was then converted into mph.

**Results:** Seventeen patients (8 M:9 F), mean age 80 ± 7.4. For all 17 patients, mobility decline was a major symptom at the point of referral. The mean pre-operative walking speed was 0.8 mph. Post-operatively this increased to 1.2 mph on average, a 50% improvement in walking speed. Of the 17 patients, only one had a deterioration in the walking speed. Pre-op average for 10 m walking test was 36 steps, and post-op was 23.

**Conclusions:** A mean improvement of 0.4 mph was observed after shunt insertion, providing further evidence that CSF diversion in INPH can effectively improve mobility.

## A12 Biomarkers in IIH: does CSF drainage influence T-tau and Aβ-42 levels?

### I. Baudracco, C. Craven, S. Matloob, S. Thompson, L. Thorne, L. D. Watkins, A. K. Toma

#### Victor Horsley Department of Neurosurgery, National Hospital for Neurology and Neurosurgery, London, UK

##### **Correspondence:** I. Baudracco

*Fluids and Barriers of the CNS* 2017, 14(Suppl 1):A12

**Introduction:** Little is known about cerebrospinal fluid (CSF) biomarkers in idiopathic intracranial hypertension (IIH). T-tau and Aβ-42 have been studied in neurodegenerative diseases. This study aimed to determine if these biomarkers levels are influenced after shunting in IIH.

**Methods:** Single-centre retrospective case series of patients, who had CSF samples taken during either: ventriculoperitoneal (VP) shunt insertion or revision or during lumboperitoneal (LP) shunt insertion or revision. An unpaired T test was used to compare the CSF biomarker levels.

**Results:** Between January 2013 to March 2016, 34 IIH patients (33 F:1 M) mean age 36.5 years, were included. The mean follow-up was 51.2 ± 23.1 months. Nineteen samples were from lumbar CSF of which 11 were primary samples and 8 during revision. Sixteen samples were ventricular CSF, of which 4 were taken during a primary VP shunt insertion and 12 were taken during a revision. The mean ventricular CSF levels were: T-tau 1240 ng/l, Aβ-42 1240 ng/l and T-tau/Aβ1-42 ratio 7.1. Mean lumbar CSF levels were: T-tau 377 ng/l, Aβ-42 1108 ng/l and T-tau/Aβ1-42 ratio 0.3. The mean ratio of T-tau/AB1-42 was significantly higher in CSF taken during shunt revision surgery compared to primary shunt insertion (p < 0.05).

**Conclusions:** Patients with IIH appear to have normal lumbar CSF levels of T-tau and Aβ-42, suggesting neurodegeneration is not a major process in the pathophysiology of the disease. Samples taken during revision surgery had, on average, higher levels of neurodegenerative markers than those taken during primary surgery, suggesting the presence of the shunt may influence the levels.

## A13 CSF outflow along all nerves: a widely unexplored aspect in the pathophysiology of neuroinflammation

### Karl Bechter

#### Department Psychiatry II/Bezirkskliniken, Ulm University, Günzburg, Germany

##### **Correspondence:** Karl Bechter

*Fluids and Barriers of the CNS* 2017, 14(Suppl 1):A13

**Introduction:** Although first described by HI Quincke in 1872 (Bechter & Benveniste 2015 NPBR) the physiological outflow of CSF along all brain nerves and peripheral nerves remained unexplored with regard to a possible role in pathophysiology in general and specifically in neuroinflammation.

**Methods:** Based on clinical observations during experimental treatments of therapy resistant psychiatric disorders with CSF filtration, shown effective for in autoimmune-neuroinflammatory disorder Guillain–Barre-syndrome, beyond the observed improvement of depression (associated with neuroinflammation) also minor peripheral neurological symptoms improved in parallel (Bechter et al. 2004, Bechter 2007). On this background and extensive literature the Peripheral CSF Outflow (PCOP)-Hypothesis was developed, suggesting a broad role for CSF signaling at the PCOP (Bechter 2011).

**Results:** With MRI the CSF outflow at the PCOP can be easily visualized (Bechter &Schmitz Croat Med J 2014). With case analysis including histopathology after death from chronic leukemia, we for the first time demonstrated, that CSF cells follow the PCOP (Schmitt et al. Anticancer Res 2011). These findings match with recent knowledge about the afferent and efferent immunological pathways of the brain (Carare et al. BBI 2014). Beyond, CSF signaling at the PCOP may result in retrograde effects in neurons, e.g. synaptic stripping, but may also modulate neuronal guided immune responses (Bechter NPBR 2016).

**Conclusions:** The predictions of the PCOP-Hypothesis remain yet poorly explored as far as investigated were confirmed. Further research in clinical and experimental approaches is required.

## A14 Changes in brain tissue mechanical properties during hydrocephalus development in adult and young rats are different

### A. C. Pong^1,2^, L. Jugé^1,2^, L. E. Bilston^1,3^, S. Cheng^1,4^

#### ^1^Neuroscience Research Australia, Randwick, Australia; ^2^School of Medical Sciences, University of New South Wales, Kensington, Australia; ^3^Prince of Wales Clinical School, University of New South Wales, Kensington, Australia; ^4^Department of Engineering, Faculty of Science and Engineering, Macquarie University, Sydney, Australia

##### **Correspondence:** L. E. Bilston

*Fluids and Barriers of the CNS* 2017, 14(Suppl 1):A14

**Introduction:** Brain stiffness increases during ventricular enlargement in juvenile animals with unfused cranial sutures (Jugé et al. 2016), however it is not clear whether this also occurs in adult animals with a fused skull. The aims of this study are to determine how brain tissue stiffness changes in adult hydrocephalic rats with a fused skull, and how these changes are related to tissue deformation.

**Methods:** Hydrocephalus was induced in nine adult Sprague–Dawley rats by injecting a kaolin suspension into the cisterna magna. Six sham-injected rats were used as controls. Anatomical MR images and MR elastograms (800 Hz) were acquired 1 day before and 3 days post injection (9.4T, Bruker). Results were also compared with existing data from young hydrocephalic rats.

**Results:** When the ventricles enlarged in adult hydrocephalic rats, brain tissue area decreased (GEE, p < 0.01) while the cranial area and cortical stiffness did not change (GEE, p = 0.40, p = 0.24, respectively). However, while the ventricles enlarged more in young hydrocephalic rats than in adult hydrocephalic rats (t test, p = 0.01), brain tissue area normalized to baseline was significantly lower (t test, p = 0.0002) in the adult hydrocephalic rats than in young hydrocephalic rats, suggesting higher brain compression in adults. Plotting stiffness against changes in normalised brain cross sectional area for both adult and young hydrocephalic rats, cortical stiffness was higher when normalized brain tissue area was lower (Spearman, p = 0.001).

**Conclusions:** This study showed that brain stiffness was higher in the more compressed adult hydrocephalic brains than the less compressed juvenile brains.

## A15 Late adult onset aqueductal stenosis may become symptomatic due to deep white matter ischemia

### W. Bradley

#### Department of Radiology, University of California San Diego Health System, San Diego, CA, USA

##### **Correspondence:** W. Bradley

*Fluids and Barriers of the CNS* 2017, 14(Suppl 1):A15

**Introduction:** While most aqueductal stenosis (AS) presents in infancy, some presents in late adulthood with a similar clinical triad as NPH—plus chronic headaches. The goal of this study was to consider a possible mechanism for a late adult onset presentation. Specifically, we believe that AS forces some CSF to exit via the extracellular space (ECS) of the brain via the glymphatic system—similar to children with tectal gliomas. With aging comes deep white matter ischemia (DWMI) which consists of myelin pallor and greater attraction between the exiting polar water molecules and the bare myelin protein, increasing resistance to CSF outflow, leading to back up of CSF both in NPH and AS.

**Methods:** Retrospective review of medical records revealed 15 elderly patients referred for possible NPH who had aqueductal webs on midsagittal, bright CSF, submillimeter CISS or FIESTA sequences. Routine MRI including DWI with ADC was performed. Age matched controls were imaged using the same routine technique.

**Results:** The apparent diffusion coefficient (ADC) was significantly higher for a given degree of DWMI for the AS patients (p < 0.018 for mild DWMI; p < 0.001 for moderate to severe DWMI) than the controls implying a higher water content in the ECS of the former.

**Conclusions:** The greater resistance to egress of CSF via the extracellular space of the brain (glymphatic system) due to DWMI may contribute to symptom onset in late adult onset AS.

## A16 Atrophy or normal pressure hydrocephalus?

### F. Hakim^1^, J. F. Ramón^1^, M. F. Cárdenas^1^, J. S. Davidson^1^, C. García^1^, D. González^1^, S. Bermúdez^2^, N. Useche ^2^

#### ^1^Department of Surgery, Section of Neurosurgery, Fundación Santa Fe de Bogotá, Bogotá, Colombia; ^2^Department of Diagnostic Imaging, Section of Neuroradiology, Fundación Santa Fe de Bogotá, Bogotá, Colombia

##### **Correspondence:** M. F. Cárdenas

*Fluids and Barriers of the CNS* 2017, 14(Suppl 1):A16

**Introduction:** The proper diagnosis of normal pressure hydrocephalus has been a matter of debate since Dr. Hakim first described it in 1964. Along with the clinical triad and the tap test, the diagnostic confirmation has been achieved with radiological findings suggesting NPH. The current radiological tools, namely CT and MRI has permitted a clearer approximation to NPH, and enabled better differentiation from other disease processes coursing with ventriculomegaly, by showing a disproportionate ventricular system size, as compared to peripheral cortical subarachnoid space. NPH tends to be subdiagnosed, with an ensuing unfavorable management strategy. This is probably due to the shared clinical and imaging characteristics between NPH and dementias, principally of the Alzheimer’s type.

**Methods:** We compared pre-operative and post-operative head CT scan cuts of five patients with NPH, treated with ventriculo-atrial shunt placement, in order to determine changes in depth, antero-posterior segment length, and area of the Sylvian Fissure.

**Results:** Analysis showed a bilateral, statistically significant reduction in the antero-posterior segment length and area of the Sylvian Fissure after shunt placement. Depth also decreased, but the difference was not statistically significant.

**Conclusions:** These post-operative changes are an indirect index of parenchymal recuperation not previously measured in an objective manner. These findings support the hypothesis that at least a proportion of patients diagnosed with cerebral atrophy, could be coursing with reversible subarachnoid space augmentation associated with NPH.

## A17 Normal pressure hydrocephalus clinical care program

### F. Hakim, J. F. Ramon, M. F. Cárdenas, J. S. Davidson, J. A. Mejía, P. Mayorga, F. Cruz, M. Mora, C. Martinez, M. C. Matiz, M. Vallejo, K. Ghotme, H. A. Soto, D. Riveros, A. Buitrago, M. Mora, L. Murcia, S. Bermudez, N. Useche, D. Cohen

#### Grupo de Hidrocefalia con Presión Normal, Hospital Universitario Fundación Santa Fe de Bogotá, Bogotá, Colombia

##### **Correspondence:** M. F. Cárdenas

*Fluids and Barriers of the CNS* 2017, 14(Suppl 1):A17

**Summary:** Despite normal pressure hydrocephalus (NPH) was described in 1964, its physiopathology has not yet been completely elucidated. Additionally, NPH diagnostic criteria and management clinical guidelines currently rely on epidemiological evidence and clinical experience. Unfortunately, epidemiological data display high variability due to the heterogeneity of diagnostic techniques and management approaches in different studies. This appears to lead to misdiagnosis and consequently to inaccurate treatment, as well as to underestimation of the number of NPH cases. There is as yet no consensus on the NPH patient´s interdisciplinary care protocol. In order to approach this limitation, a NPH Clinical Care Program was established at Hospital Universitario Fundación Santa Fe de Bogotá (FSFB) in 2014. The main objective of the program is to identify aspects that can be intervened in order to modify the course of the disease. This program consists as an interdisciplinary group of health care professionals, led by the Neurosurgery Department, and aims at designing and establishing of an assertive and early NPH detection for an optimal case management. The current approach of the FSB NPH-Program consists in offering early diagnosis; prompt treatment and initially a 1-year of patient’s follow up The program has the final goal of improving the quality of NPH attention that may allow patients to be comfortable and confident in daily life with their families and in the community, and consequently a more effective health care. We will present the experience of the FSB NPH-Program and the importance of an accurate diagnose and treatment.

## A18 Ventricular T-tau and Aβ-42 levels in 78 shunted INPH patients

### C. Craven, I. Baudracco, S. Matloob, S. Thompson, L. Thorne, A. K. Toma, L. D. Watkins

#### Victor Horsley Department of Neurosurgery, National Hospital for Neurology and Neurosurgery, London, UK

##### **Correspondence:** C. Craven

*Fluids and Barriers of the CNS* 2017, 14(Suppl 1):A18

**Introduction:** Idiopathic normal-pressure hydrocephalus (INPH) has no reliable biomarkers that assist in the selection of patients for a ventriculoperitoneal (VP) shunt. T-tau and Aβ-42 have been implicated as biomarkers in other neurodegenerative diseases and in INPH. The aim of this study was to review their ability to predict outcome after VP shunting.

**Methods:** Single-centre retrospective analysis of probable INPH patients with ventricular CSF samples. Samples were sent for ELISA analysis. Clinical documentation was reviewed for outcome. An unpaired T test was used to compare the CSF biomarker levels. Negative and positive predictive values (NPV and PPV) for shunt-responsiveness were calculated.

**Results:** August 2006 to July 2015, 78 (31 F:47 M) INPH patients, mean age 75.3 (R 55–94) had ventricular CSF analysed during shunt insertion or revision. Mean follow-up was 959 ± 657 days. Mean CSF levels for T-tau, Aβ-42 535 and T-tau/Aβ1-42 ratio were 669 ng/l, 471 ng/l and 2.00 respectively. There was no significant difference in levels between the shunt responsive and non-responsive group (p > 0.05). The markers demonstrated poor predictive value for shunt responsiveness (PPV 59% and NPV 41%). There was no significant difference in T-tau/Aβ1-42 ratio between those with INPH and a neurodegenerative diagnosis (mainly Alzheimer’s and Parkinson’s disease) compared to those with probable INPH alone. Eleven of fourteen with a neurodegenerative diagnosis were still shunt responsive.

**Conclusions:** Neither T-tau nor Aβ-42 were effective at predicting shunt responsiveness in this group. Biomarkers more specific to INPH, with an ability to predict shunt responsiveness still need to be identified.

## A19 Morphology of dural venous sinus stenosis in IIH

### C. Craven, D. Dasgupta, S. Matloob, S. Thompson, I. Baudracco, L. Thorne, A. K. Toma, L. D. Watkins

#### Victor Horsley Department of Neurosurgery, National Hospital for Neurology and Neurosurgery, London, UK

##### **Correspondence:** D. Dasgupta

*Fluids and Barriers of the CNS* 2017, 14(Suppl 1):A19

**Introduction:** Idiopathic intracranial hypertension (IIH) is commonly associated with venous sinus stenosis and dural venous sinus stenting (DVSS) is becoming an increasingly used treatment option. Dural venous stenosis can be broadly classified into extrinsic (extra-luminal) or intrinsic (intra-luminal due to a thrombus or arachnoid granulation). We aimed to determine if morphological differences in sinus stenosis affected clinical or radiological outcome.

**Methods:** A single centre case series. Clinical outcomes were assigned using degree of papilloedema on fundoscopy as a surrogate measure. Angiographic and manometry data before stenting and at 3 months after stent placement were reviewed. Differences in clinical outcomes, and development of re-stenosis in the two groups were assessed using Wilcoxon matched-pairs signed rank test and Fisher’s exact test (Chi squared) respectively.

**Results:** Between September 2010 and March 2016, 41 patients (2 M:39 F), mean age of 35.7 underwent DVSS, of whom 24 had an extrinsic and 17 had an intrinsic stenosis within the 3-month follow-up period. Sixteen of the 24 patients with an extrinsic stenosis developed a further morphological stenosis, compared to three within the intrinsic stenosis group (p < 0.05). There was no significant difference in development of re-stenosis with a significant pressure gradient (p = 0.12).

**Conclusions:** Extrinsic venous sinus stenosis is more common radiologically and more likely to result in a re-stenosis post stenting. However there was no increase in re-stenosis with a significant pressure gradient, nor difference in outcome on fundoscopy. The high proportion of re-stenosis occurring those with extrinsic stenosis may provide further insight into the pathophysiology of IIH.

## A20 Rostral-caudal CSF markers of inflammation and infection in SAH patients

### C. Craven, I. Baudracco, S. Matloob, S. Thompson, L. D. Watkins, A. K. Toma

#### Victor Horsley Department of Neurosurgery, National Hospital for Neurology and Neurosurgery, London, UK

##### **Correspondence:** C. Craven

*Fluids and Barriers of the CNS* 2017, 14(Suppl 1):A20

**Introduction:** Acute hydrocephalus is a common complication of subarachnoid haemorrhage (SAH). Sampling of cerebrospinal fluid (CSF) from a pre-existing drain [external ventricular drain (EVD) or lumbar drain (LD)] is commonly performed to rule out infection prior to ventriculoperitoneal (VP) shunt insertion. Study aims to determine (1) if lumbar CSF infection markers reflects that of ventricular CSF (2) if abnormal WCC in LD correlated with a subsequent shunt infection.

**Methods:** Single centre analysis of CSF from lumbar and ventricular CSF, sampled during the removal of LD and insertion of an EVD or VP shunt. Simultaneous CSF samples were sent for culture, WCC and biochemical analysis. A paired T test was performed to determine if levels were significantly different in lumbar and ventricular samples. An F-test compared the variances.

**Results:** Five patients (4 M:1F), aged 67 ± 13.44 (mean ± SD), had hydrocephalus secondary to SAH. Lumbar CSF samples had a mean WCC of 939 ± 1894 cu/mm, ventricular CSF had a mean 57.0 ± 105 cu/mm. Lumbar samples had a total protein of 3.80 ± 3.41 g/l, greater than ventricular samples level of 1.76 ± 2.09 g/l. Lumbar WCC levels had a significantly higher variance (p < 0.05). No patients developed a subsequent VP shunt infection.

**Conclusions:** There is a rostral-caudal disparity in WCC and total protein levels, with lumbar CSF having greater WCC and total protein levels than ventricular CSF taken simultaneously. Decision to proceed to shunt insertion should be guided by clinical picture rather than cell count on thecal CSF.

## A21 Simulation workshops and standardised care bundles improve external ventricular drain placement and decrease infections

### D. Dasgupta^1^, A. K. Toma^1^, C. Curtis^2^, L. D. Watkins^1^, L. Thorne^1^

#### ^1^Victor Horsley Department of Neurosurgery, National Hospital for Neurology and Neurosurgery, London, UK; ^2^Department of Microbiology, University College London Hospital NHS Foundation Trust, London, UK

##### **Correspondence:** D. Dasgupt

*Fluids and Barriers of the CNS* 2017, 14(Suppl 1):A21

**Introduction:** Temporary cerebrospinal fluid (CSF) diversion through an external ventricular drain (EVD) comes with the risk of EVD-related infections (ERIs)—incidence varies from 0.8 to 22%. ERIs increase mortality, length of stay, costs, prolonged courses of antibiotics, and increase subsequent permanent CSF diversion. The rationale for this quality improvement project was therefore to reduce infection rates and improve placement via simulation training and a standardised perioperative care bundle.

**Methods:** A best practice standardised perioperative approach and care bundle was peer-agreed amongst the senior neurosurgeons at the National Hospital for Neurology and Neurosurgery, Queen Square, London, and a standardised operation note was designed to improve documentation, and involve carers in positive decision-making. This was adapted from the bundle by Kubilay et al. Targeted simulation workshops included safe access, administration of intrathecal drugs, and demonstration of safe operative technique using a perpendicular trajectory using the Rowena head. Standardised aspects of care included choice of catheter, length of tunnelling, and method of securing the drain.

**Results:** The infection rate before implementation was 5% (n = 17), placement was satisfactory in 50% (n = 16), and mean length of drainage was 8 days. In the 6 months following the interventions, preliminary results suggest the infection rate was 1.4% (n = 70), placement was satisfactory in 86.7% (n = 60), and mean length of drainage was 9 days.

**Conclusions:** This demonstrates that simulation training and standardising perioperative care of patients requiring EVDs dramatically reduces infection rates and accuracy of placement, resulting in improving patient outcomes and reducing length of stay.

## A22 Role of endoscopic third ventriculostomy in the management of myelomeningocele related hydrocephalus. 16 case series in Cartagena de Indias: Colombia

### L. Domínguez, A. J. Remolina, M. A. Grijalba

#### Neurosurgery Department, Cartagena University, Cartagena de Indias, Colombia

##### **Correspondence:** L. Domínguez

*Fluids and Barriers of the CNS* 2017, 14(Suppl 1):A22

**Introduction:** Hydrocephalus (HC) treatment, related to myelomeningocele, through endoscopic third ventriculostomy (TVE) is controversial because of the fact that traditionally, ventriculoperitoneal shunt (VPS) represent a principal treatment for most of the patients. Consequently, our experience in the pathology treatment through TVE is presented.

**Materials and methods:** A retrospective description was made for every patient, which was subjected to TVE to treat hydrocephalus related to Myelomeningocele (MMC) between January 2013 and May 2016.

**Results:** 15/16 patients had previous VPS, debuted with symptoms and signs of system dysfunctions and one case with intracranial hypertension syndrome (IHS) anew. 15/16 patients showed clinical and radiological improvement post TVE and in just one case IHS symptoms recurred.

**Conclusions:** Hydrocephalus is the principal problem for patients with MMC, which develops in up to 80–90%, after spinal defect closure. The VPS, at present the principal option for treatment, however, is related to high morbidity in this population. In spite of bad outcome a few years ago, and the difficulties TVE represents because of anatomical variations and neurological immaturity of the cerebrospinal fluid (CSF) system in these patients, highly qualified personnel recognize that in children after 6 months old, TVE is a valuable option for HC treatment associated with MMC.

In our experience, the TVE in pediatric patients with MMC has a high level of success (93.7%) made in well-chosen patients.

## A23 “I’m referring a patient with a shunt…” Diagnostic complexities of CSF shunt complications in children

### K. J. Whitehouse, R. J. Edwards

#### Department of Paediatric Neurosurgery, Bristol Royal Hospital for Children, Bristol, UK

##### **Correspondence:** R. J. Edwards

*Fluids and Barriers of the CNS* 2017, 14(Suppl 1):A23

**Introduction:** CSF shunts have a high revision rate, but there is little in the literature about how patients referred with possible shunt failure are assessed and diagnosed.

**Methods:** All children referred to a supra-regional paediatric neurosurgical centre with suspected CSF shunt failure over 6-month period were identified on a prospective database and a casenote review evaluating the diagnostic process was undertaken.

**Results:** There were 100 emergency referrals for 61 children (mean age 6.4-years) with shunts, 46 of whom had never been revised, mean time since last shunt procedure was 3.3 years. 36 children required admission, 23 underwent revision (all requiring change of a part of the shunt at operation). 51% of referrals resulted in neurosurgical review, 9 required ventricular access and 6 required ICP monitoring. Five patients required multiple shunt operations.

A scale was developed to communicate the difficulty and level of invasiveness of investigation required to diagnose or exclude shunt failure, from 1 (not a query relating to potential blockage) to 5 (difficult to determine shunt issue requiring surgical exploration).

**Conclusions:** The proposed scale can help clinicians understand the referral patterns of patients with shunts; and convey the increasing complexity of this population and need to undertake invasive investigations to diagnose shunt blockage. It can also aid longitudinal analysis of neurosurgery unit data and identify the subgroup of patients for whom imaging studies are of low diagnostic utility, and where ventricular access device placement may useful to facilitate more invasive evaluation of shunt function such as CSF infusion studies or ICP monitoring.

## A24 Identical twins with idiopathic normal pressure hydrocephalus

### A. Eleftheriou^1^, F. Lundin^2^

#### ^1^Department of Neurology, University Hospital, Linköping, Sweden; ^2^Division of Neuroscience, Department of Clinical and Experimental Medicine (IKE), Linköping University, Linköping, Sweden

##### **Correspondence:** A. Eleftherio

*Fluids and Barriers of the CNS* 2017, 14(Suppl 1):A24

**Summary:** Idiopathic normal pressure hydrocephalus (iNPH) is usually regarded as a sporadic disease with typical gait, cognitive and urinary symptoms caused by a disturbance of the cerebrospinal fluid dynamics. The pathophysiological mechanisms of iNPH are still poorly understood. In the literature there are a few reports of familial clustering of iNPH. We present the first case of iNPH in two adult identical twins. Both developed similar symptoms at approximately the same age. Their MRI showed enlargement of the ventricles, a disproportional narrowing of the subarachnoid space and cortical sulci at the high convexity of the cerebrum and sharp callosal angle. Both of them improved significantly after shunt surgery confirmed by a 3 month-postoperative examination. This indicates a possible genetic component involved in the pathogenesis. Further genetic studies are needed in this poorly explored area.

## A25 Endoscopic third ventriculostomy in normal pressure hydrocephalus patients

### K. N. Fountas^1^, E. Z. Kapsalaki^2^, H. F. Smisson^3^, J. S. Robinson^3^

#### ^1^Department of Neurosurgery, School of Medicine, University of Thessaly, Larisa, Greece; ^2^Department of Diagnostic Radiology, School of Medicine, University of Thessaly, Larisa, Greece; ^3^Department of Neurosurgery, Georgia Neurosurgical Institute, Macon, GA, USA

##### **Correspondence:** K. N. Fountas

*Fluids and Barriers of the CNS* 2017, 14(Suppl 1):A25

**Introduction:** Surgical management of idiopathic normal pressure hydrocephalus (iNPH) remains puzzling despite the wide clinical application of programmable CSF shunts. The shunt-associated complications, along with the increased cost of the shunt, constitute the major drawbacks for shunting as a treatment for iNPH patients. Endoscopic third ventriculostomy has been employed in several small series of selected iNPH patients with variable results.

**Materials and methods:** Our prospective study included 23 patients (4 females, 19 males) with ages ranged between 63 and 76 years (mean 70.4), suffering iNPH. All participants underwent preoperatively conventional and cine-MRI study, 40–50 ml CSF drainage via a spinal tap, and neurocognitive evaluation by employing the Frontal Assessment Battery (FAB) and the Montreal Cognitive Assessment (MOCA) tests. All patients underwent an endoscopic third ventriculostomy, while another MRI study and neuropsychological evaluations were obtained 6 months postoperatively, and then at 6 months interval for up to 2 years. The idiopathic normal pressure hydrocephalus grading system (INPHGS) was also employed pre- and post-operatively in all participants. The follow up time ranged in our cohort from 6 to 96 months (mean 40.2).

**Results:** The mean postoperative INPHGS score was 5.2, while the preoperative one was 6.8 in our current series. The mean postoperative FAB score was 14.3 from 12.5, while the mean postoperative MOCA score was 24.2 from 21.5 preoperatively. The mean postoperative CSF stroke volume was 33.2 μl from 47.0 μl preoperatively.

**Conclusions:** Endoscopic third ventriculostomy seems to be a valid surgical alternative option in iNPH patients, with functional CSF obstruction.

## A26 Telemetric pressure monitoring: the role in the clinical management of hydrocephalus

### M. J. Fritsch, W. Arouk

#### Klinik für Neurochirurgie, Dietrich-Bonhoeffer-Klinikum, Neubrandenburg, Germany

##### **Correspondence:** M. J. Fritsch

*Fluids and Barriers of the CNS* 2017, 14(Suppl 1):A26

**Introduction:** One indication for the application of telemetric pressure monitoring (TPM) occurs in patients (with or without shunt) presenting with unclear clinical symptoms (e.g. headaches), not explained by imaging studies or the patients history. The question we wanted to answer is: Does this patient need treatment? Our second indication is the optimization/adjustment of a preexisting (adjustable) shunt. Here the task is: How can we adjust the valve in the best possible way.

**Methods:** Between November 2012 and October 2015 a total of 15 patients (11 male, 4 female) underwent TPM. A Raumedic sensor was implanted in 13 patients and a Miethke sensor reservoir in two patients. Age was between 4 and 35 years (mean 14 a) and the Follow up between 6 and 32 months (mean 18 mo). Our algorithm was: Implantation—Measurement at the hospital—Training (patient/family)**—**Measurement at home (2–4 weeks)—Evaluation of data—Decision.

**Results:** In all patients, pressure monitoring under daily conditions in the hospital and at home was helpful in guiding therapy decisions (implantation or revision of a shunt, decision against shunt/or any other surgery, valve adjustment, other surgeries like cranioplasty). We present several case examples.

**Conclusions:** Telemetric pressure monitoring is a helpful tool to guide clinical decision making in complex or difficult cases. TPM adds pressure data to clinical judgment and imaging studies and it provides new insight in the correlation between symptoms (e.g. headaches) and normal or pathologic ICP.

## A27 Evaluation of a new treatment protocol for hydrocephalus secondary to intraventricular haemorrhage (IVH) in preterm babies

### M. Garzon, M. Kang, K. Sandhu, D. Baghawatti, K. Aquilina, G. James, D. Thompson

#### Great Ormond Street Hospital, London, UK

##### **Correspondence:** M. Garzon

*Fluids and Barriers of the CNS* 2017, 14(Suppl 1):A27

**Introduction:** IVH is the commonest neurosurgical presentation in preterm infants. In 2012, we introduced a new protocol for its management, using ventriculo-subgaleal shunts (VSGS) as the preferred temporising device.

**Methods:** Retrospective case review on electronic records. All the preterm infants requiring VSGS between 01/12 and 01/14 were included.

**Results:** During this period, 23 infants required VSGS; mean birth-weight was 897 g. Nineteen (82.6%) presented with IVH Papile grade 3 or 4 IVH at the time of referral. At the time of VSGS insertion, mean gestational age was 31 + 1 weeks (range 26 + 4 to 36 + 4), and the weight was 1171 g (range 758–1600 g). Pre- and post- surgical ventricular indices (VI) demonstrated an average 7.6 mm reduction. Surgical complications occurred in 7 cases (30%), including 2 infections, 2 failures requiring revision, and 3 decompressive haemorrhages (managed conservatively). The mean follow up was 516 days (range 137–1056). Twenty-two infants (95.6%) underwent conversion to a permanent ventriculoperitoneal shunt, within a mean time of 79 days (range 24–349) after the VSGS insertion. Mortality was zero.

**Conclusions:** VSGS is a safe therapeutic option for preterm IVH with an acceptable morbidity profile in these complex patients. Despite its conceptual use as temporising device, all, except one patient required insertion of a permanent shunt. This may relate to the severe Papile-grade in our population.

## A28 Modelling of posture-related changes in cerebrospinal fluid dynamics

### M. Gehlen^1,2^, V. Kurtcuoglu^2,3^, M. Schmid Daners^1^

#### ^1^Department of Mechanical and Process Engineering, ETH Zurich, Zurich, Switzerland; ^2^Institute of Physiology, University of Zurich, Zurich, Switzerland; ^3^Neuroscience Center Zurich, and the Zurich Center for Integrative Human Physiology, University of Zurich, Zurich, Switzerland

##### **Correspondence:** M. Gehlen

*Fluids and Barriers of the CNS* 2017, 14(Suppl 1):A28

**Introduction:** Postural changes lead to changes in intracranial pressure, but also to a shift in the cranio-spinal compliance distribution of the cerebrospinal fluid (CSF) system. We hypothesize that this shift is caused by the collapse of the jugular veins and aim to test the hypothesis with a lumped-parameter model of CSF dynamics.

**Methods:** An exponential function was used to describe the relationship between CSF pressure—relative to venous pressure—and volume. 63% of this CSF-to-venous compliance was assumed to be located spinally. The hydrostatic pressure column in the CSF system was assumed to be uninterrupted in upright posture, while the one in the veins was assumed to be interrupted at the jugular veins. The venous pressure at the spinal level was modelled as posture independent.

**Results:** Reaching the same overall compliance in supine and upright equilibrium required 22% of the CSF absorption to be located spinally. With this, the overall CSF volume in upright equilibrium was increased by 1.6 ml. Sitting up caused an immediate caudal CSF shift of 2.5 ml and a spinal compliance decrease from 63 to 22% of the overall value. Due to this shift in compliance, the ability to compensate for cerebral arterial volume pulsations was reduced and the cranio-spinal CSF flow pulsations decreased from 328 to 61 ml/min peak-to-peak amplitude.

**Conclusions:** The good accordance of the here modelled changes with values observed in vivo lead us to the conclusion that the jugular collapse is a major contributor to the posture-related changes in CSF dynamics.

## A29 In vitro comparison of anti-siphon mechanisms under postural changes

### M. Gehlen^1,2^, A. Eklund^3^, V. Kurtcuoglu^2,4^, J. Malm^5^, M. Schmid Daners^1^

#### ^1^Department of Mechanical and Process Engineering, ETH Zurich, Zurich, Switzerland; ^2^Institute of Physiology, University of Zurich, Zurich, Switzerland; ^3^Department of Radiation Sciences, Umeå University, Umeå, Sweden; ^4^Neuroscience Center Zurich, and the Zurich Center for Integrative Human Physiology, University of Zurich, Zurich, Switzerland; ^5^Department of Pharmacology and Clinical Neuroscience, Umeå University, Umeå, Sweden

##### **Correspondence:** M. Gehlen

*Fluids and Barriers of the CNS* 2017, 14(Suppl 1):A29

**Introduction:** Flow-regulated, gravitational, and membrane-controlled anti-siphon devices (ASD) have been developed to counteract siphoning-induced overdrainage in upright posture through cerebrospinal fluid (CSF) shunts. We aimed to elucidate how these three types of ASDs interact with the CSF system under postural changes.

**Methods:** Three shunts each of Codman Hakim with SiphonGuard (flow-regulated), Miethke miniNAV with proSA (gravitational), and Medtronic Delta (membrane-controlled) were tested. The pressure-flow characteristic of each shunt was quantified in terms of opening pressure, closing pressure, and resistance. CSF drainage rates, resulting CSF volume change and intracranial pressure (ICP) were measured in supine, sitting, and standing posture. The measurements were performed on a robotic test bench that models the in vivo environment of the shunt based on a mathematical description of CSF dynamics.

**Results:** The flow-regulated ASDs avoided increased drainage in sitting posture, but reopening of the ASD was observed while standing. This reopening may be problematic for short patients and patients with increased IPP. The gravitational ASDs allowed setting the opening pressures in horizontal and vertical orientation independently. However, as their drainage rate changes with IPP, uniform drainage in upright posture is impossible and adaptation to the patient is critical. The membrane-controlled ASDs eliminated overdrainage by stopping drainage in upright posture at the expense of CSF accumulation.

**Conclusions:** All tested ASDs reduced overdrainage, but their effects on CSF dynamics varied greatly: While membrane-controlled ASDs are a robust means of siphon-prevention, flow-controlled devices provide continuous drainage, and gravitational ASDs allow patient-specific adaptation, but precise adjustment is required.

## A30 Ventriculoatrial shunt as the first option: our experience in the management of hydrocephalus in adults

### F. Hakim, J. F. Ramon, D. Gomez

#### Neurosurgery Department, “Hospital Universitario, Fundación Santafe de Bogota”, Bogota, Colombia

##### **Correspondence:** D. Gomez

*Fluids and Barriers of the CNS* 2017, 14(Suppl 1):A30

**Introduction:** Ventricular shunts represent the axis of treatment of disorders of the cerebrospinal fluid (CSF). The literature shows that the preferred technique around the world is the ventricular peritoneal shunt (VP), which is technically more simple, reproducible and represents a permanent and lasting CSF drainage. In our institution ventriculoatrial derivation is the first choice in the treatment of hydrocephalus in adults, results are equivalent to the technique of choice or even better.

**Methods:** A retrospective review of patients with hydrocephalus who were treated with ventriculoatrial shunting at a single institution, from 2007 to 2015, was performed.

**Results:** The results will be shown at the meeting.

**Conclusion:** Ventriculoatrial shunt is a valid option in the management of hydrocephalus, in our experience is a safe, reproducible and effective technique. We consider that a DVA shunt is more physiological than traditional techniques and less shunt malfunction in the follow up.

## A31 Encapsulation of subcommissural organ (SCO) cells to promote neurogenesis in foetal onset hydrocephalus

### M. Guerra^1^, M. Jara^1^, M. Flores^2^, K. Vío^1^, I. Moreno^2^, S. Rodríguez^1^, E. Ortega^3^, E. M. Rodríguez^1^

#### ^1^Instituto de Anatomía, Histología y Patología, Facultad de Medicina, UACh, Valdivia, Chile; ^2^Laboratorio de Polímeros, Facultad de Ciencias, UACh, Valdivia, Chile; ^3^Instituto de Neurociencias Clínicas, Facultad de Medicina, UACh, Valdivia, Chile

##### **Correspondence:** M. Guerra

*Fluids and Barriers of the CNS* 2017, 14(Suppl 1):A31

**Introduction:** Prenatal and adult neurogenesis is impaired in hydrocephalus. The aim of the present investigation was to encapsulate cells of the subcommissural organ (SCO) in order to develop a neurotrophic therapy for hydrocephalic children. SCO cells secrete SCO-spondin, transthyretin, fibroblast growth factor, and S100β. These compounds have neurotrophic, neuroprotective and immunomodulatory functions [1].

**Methods:** SCO explants from bovine were maintained in organ culture for 30 days, a time when they become secretory ependymospheres. These spheres were encapsulated by using different polymeric semipermeable membranes. Encapsulated ependymospheres were cultured for 6 months with complete culture medium containing DMEM/F12HAM supplemented with 10% fetal calf serum and 5% bovine serum. Double immunofluorescence was used to analyze the phenotype of SCO-cells after encapsulation and a long term in culture. Western blotting analyses were performed to study the presence of SCO-secretory compounds in the conditioned medium. Culture of rat neural stem cells (NSC) or human SH-SY5Y cells in the presence of encapsulated ependymospheres or of their condition medium was used as an assay to evaluate neurogenetic properties of SCO.

**Results:** (I) SCO-encapsulated cells maintained their phenotype and they keep on secreting neurotrophic factors to the conditioned media for at least 6 months; (II) SCO-spondin was detected in the conditioned media of encapsulated cells; (III) In the differentiation assay, NSCs and SH-SY5Y cells differentiated into neurons.

**Conclusions:** Encapsulated-SCO may become a useful tool to promote neuronal differentiation in the brain. Since xenografting of encapsulated tissue does not trigger a host-versus-graft reaction, grafting of encapsulated bovine SCO into the hydrocephalic cerebrospinal fluid may become a fruitful clinical tool. This would provide an opportunity for the early treatment of the neurological impairment associated with the onset fetal hydrocephalus.

**Reference**

1.: Guerra M, González C, Caprile T, Jara M, Vío K, Muñoz RI, Rodríguez S, Rodríguez EM. Understanding how the subcommissural organ and other periventricular secretory structures contribute via the cerebrospinal fluid to neurogenesis. Front Cell Neurosci. 2015;9:480.

## A32 Ventricular zone disruption in patients with post-hemorrhagic hydrocephalus of prematurity

### J. P. McAllister^1^, M. M. Guerra^3^, E. M. Rodriguez^3^, D. M. Morales^1^, D. Sival^4^, A. Jimenez^5^, D. D. Limbrick^1,2^

#### ^1^Department of Neurosurgery, St. Louis Children’s Hospital, St. Louis, MO, USA;^2^Department of Pediatrics, St. Louis Children’s Hospital, St. Louis, MO, USA; ^3^Instituto de Histologia y Patologia, Facultad de Medicina, Universidad Austral de Chile, Valdivia, Chile; ^4^Department of Pediatrics Pathology and Medical Biology, University Medical Center Groningen, University of Groningen, Groningen, The Netherlands; ^5^Departamento de Biología Celular, Genética y Fisiología Facultad de Ciencias, Universidad de Malaga, Malaga, Spain

##### **Correspondence:** M. M. Guerra

*Fluids and Barriers of the CNS* 2017, 14(Suppl 1):A32

**Introduction:** Experimental and clinical studies have documented disruption of the ventricular (VZ) and subependymal (SVZ) zones in fetal-onset congenital hydrocephalus [1–3], but no studies have included patients with hydrocephalus following intraventricular hemorrhage (IVH). To test the hypothesis that VZ and SVZ alterations are associated with IVH, immunohistochemistry was performed on tissue from neonates with this condition.

**Methods:** IVH cases (n = 15) were classified using Papile’s ultrasound-based grading system [4] and compared to controls (n = 4) with no hemorrhage or ventriculomegaly who expired from non-neurological causes. Specimens were matched by estimated gestational age (EGA). Postmortem tissue from frontal cortical and subcortical regions was processed by routine histology and immunohistochemistry for glial fibrillary acidic protein (GFAP for stem cells and mature astrocytes), β-3 tubulin (for neural progenitors), β-4 tubulin (for ciliated ependymal cells), aquaporin-4 (AQP4 for ependyma and astrocytes), and L1-cell adhesion molecule.

**Results:** The ranges of patient birth and expiration age were 23.0–39.1 and 23.7–44.1 weeks EGA, respectively; survival was 0–42 days (median 2.0 days). IVH grade did not correlate with ventricular size. All control cases exhibited normal VZ/SVZ cytology, i.e. GFAP-positive and β-4 tubulin-positive cells lining the ventricles and abundant β-3 tubulin-positive neural progenitors. All IVH cases exhibited multiple sites of parenchymal hemorrhage (including the ganglionic eminence), patchy loss of ciliated ependymal cells and stem cell processes, and periventricular heterotopias that were not correlated with IVH grade. AQP4 appeared in the plasma membrane of neural stem cells, ependyma cells and on SVZ astrocytes. GFAP-positive cells appeared to replace ependymal cell gaps in longer-term survivors.

**Conclusions:** These findings indicate that VZ/SVZ disruption occurs consistently in neonates with IVH and hydrocephalus and increases understanding of the pathogenesis of this disorder.

**References**

1.: Sival DA, et al. Neuroependymal denudation is in progress in full-term human foetal spina bifida aperta. Brain Pathol. 2011;21(2):163–79.
2.: Rodriguez EM, et al. A cell junction pathology of neural stem cells leads to abnormal neurogenesis and hydrocephalus. Biol Res. 2012;45(3):231–42.
3.: Guerra M, et al. A cell junction pathology of neural stem cells is associated to ventricular zone disruption, hydrocephalus and neurogenesis abnormalities. J Neuropathol Exp Neurol. 2015;74(7):653–71.
4.: Burstein J, Papile LA, Burstein R. Intraventricular hemorrhage and hydrocephalus in premature newborns: a prospective study with CT. AJR Am J Roentgenol. 1979;132(4): 631–5.

## A33 Early and delayed quantitative assessments on tap test for diagnosis of idiopathic normal pressure hydrocephalus

### M. Ishikawa^1,2^, S. Yamada^2,3^, K. Yamamoto^3^

#### ^1^Rakuwa Villa Ilios, Kyoto, Japan; ^2^Normal Pressure Hydrocephalus Center, Otowa Hospital, Kyoto, Japan; ^3^Department of Neurosurgery, Otowa Hospital, Kyoto, Japan

##### **Correspondence:** M. Ishikaw

*Fluids and Barriers of the CNS* 2017, 14(Suppl 1):A33

**Introduction:** Idiopathic normal pressure hydrocephalus (iNPH) is a disorder of gait, cognition and urination in the aged population. Cerebrospinal fluid (CSF) tap test is useful for diagnosis of iNPH, but its diagnostic accuracy was relatively low. To improve the accuracy, we performed early and delayed quantitative assessments for gait on day 1 and day 4.

**Methods:** Fifty-seven patients who were treated with CSF shunt surgery were subjected in this study. Assessments included 3-m timed up and go test (TUG) and 10-m walk by time and step. The values of area under the curve (AUC), sensitivity, specificity and cutoff were computed using receiver-operating curves.

**Results:** On all three examinations, improvement of gait on day 4 was same as or more frequent than that on day 1. High AUC values above 0.75 were noted in TUG and TUG (%) on day 1, and 10-m walk (step) and 10-m walk (% time) on day 4. Among them, percent change of TUG on day 1 showed highest AUC of 0.784 and cutoff was 9.3% (sensitivity 83%, specificity 78%). On day 4, AUC values on TUG and 10-m walk were comparable to those on day 1, but statistical differences were not noted. We made a subgroup of better values of variables between day 1 and day 4. They showed high AUC values, but no statistical differences were noted.

**Conclusions:** Percent change of TUG on day 1 would be favorable for predicting the effectiveness of shunt surgery.

## A34 Health-related quality of life outcome in patients with idiopathic normal pressure hydrocephalus: a 1-year follow-up study

### A. Junkkari^1^, A. Häyrinen^1^, T. Rauramaa^2^, H. Sintonen^3^, O. Nerg^4^, A. M. Koivisto^4^, R. P. Roine^5^, H. Viinamäki^6^, H. Soininen^7^, A. Luikku^4^, J. E. Jääskeläinen^1^, V. Leinonen^1^

#### ^1^Neurosurgery of NeuroCenter, Kuopio University Hospital and University of Eastern Finland, Kuopio, Finland; ^2^Department of Pathology, Kuopio University Hospital and University of Eastern Finland, Kuopio, Finland; ^3^Department of Public Health, University of Helsinki, Helsinki, Finland; ^4^Neurology of NeuroCenter, University of Eastern Finland and Kuopio University Hospital, Kuopio, Finland; ^5^University of Eastern Finland, Kuopio Finland and Helsinki and Uusimaa Hospital District, Group Administration, Helsinki, Finland; ^6^Department of Psychiatry, Kuopio University Hospital and University of Eastern Finland, Kuopio, Finland; ^7^Department of Neurology, University of Eastern Finland, Kuopio, Finland

##### **Correspondence:** A. Junkkari

*Fluids and Barriers of the CNS* 2017, 14(Suppl 1):A34

**Introduction:** This prospective study explored the factors affecting the health-related quality of life (HRQoL) outcome in patients with idiopathic normal pressure hydrocephalus (iNPH) 1 year after the installation of the cerebrospinal fluid (CSF) shunt.

**Methods:** HRQoL outcome was evaluated using 15D instrument, in which the minimum clinically significant change/difference has been estimated to be ±0.015. The follow-up data (15D, Mini-Mental State Examination, Beck Depression Inventory, iNPH Grading Scale), frontal cortical biopsy, Charlson Age Comorbidity Index and body mass index (BMI) of 145 patients diagnosed with iNPH by clinical and radiological examination were analyzed.

**Results:** At 1 year follow-up 63 (43%) patients had experienced a clinically significant improvement in HRQoL. Multivariate binary logistic regression analysis indicated that the absence of Aβ and HPτ pathology in the frontal cortical biopsy (53 vs. 33%, absolute risk difference, 20%; adjusted OR = 2.27, 95% CI 1.07–4.84; p < 0.05) and lower BMI (adjusted OR = 0.90, 95% CI 0.82–0.98; p < 0.05) predicted favorable HRQoL outcome 1 year after the shunting.

**Conclusions:** Less than half of the patients with iNPH experienced clinically significant favorable HRQoL outcome, partly explained by the patient’s characteristics and comorbidities. The HRQoL approach reveals aspects that are important for the patient’s well-being, but may also improve the quality of the outcome assessment of CSF shunting. Study results may help clinicians to estimate which patient will benefit shunt surgery.

## A35 Normal pressure hydrocephalus: is it worthwhile to treat bedridden patients?

### U. Kehler

#### Neurosurgical Department, Asklepios Klinik Hamburg Altona, Hamburg, Germany

##### **Correspondence:** U. Kehler

*Fluids and Barriers of the CNS* 2017, 14(Suppl 1):A35

**Introduction:** Normal pressure hydrocephalus (NPH) is a progressive disease. If not treated in time, patients end bedridden with total incontinence and severe dementia. The more advanced the disease is the worse the outcome of shunting. This is often the reason to refuse surgery in bedridden patients. The aim of this work is to show if bedridden patients with NPH still can benefit from surgery.

**Methods:** We treated five bedridden patients with NPH in the last 3 years. They had all the diagnostic standard work-up with clinical examination, MRI-imaging and repeated spinal tap test. The evaluation of STT was done by clinical impression of the movement improvement: the bedridden patients were asked to sit at the bedside. The effectiveness to realize this position change was evaluated as well as video-recorded in the last patients.

**Results:** Five bedridden patients with strong suspicion of NPH were evaluated with STT. All showed slight improvement although none was able to get to the sitting position by himself. However, all patients got back to ambulating stage (1 independent, 4 with walking aids) after shunting.

**Conclusions:** These five cases prove that despite the advanced stages of NPH, treatment can be effective and can improve substantially quality of life. This means that bedridden patients should not be excluded from NPH-therapy. However, evaluation criteria should be developed to help in the decision making of shunting these patients. Video recording of patient’s movements before and after spinal tap test or lumbar drainage may be helpful to detect improvements, and can be discussed with the treatment team as well as relatives to indicate shunt surgery.

## A36 The symptoms of hakim’s triad in relation to morphological findings in neuroradiology

### O. Lilja-Lund^1^, K. Kockum^1^, E. M. Larsson^2^, K. Riklund^3^, L. Söderström^1^, P. Hellström^4^, K. Laurell^1^

#### ^1^Department of Pharmacology and Clinical Neuroscience, Unit of Neurology, Östersund, Umeå University, Umeå, Sweden; ^2^Department of Radiology, Uppsala University, Uppsala, Sweden; ^3^Department of Radiation Sciences, Umeå University, Umeå, Sweden; ^4^Hydrocephalus Research Unit, Institute of Neuroscience and Physiology, The Sahlgrenska Academy, University of Gothenburg, Gothenburg, Sweden

##### **Correspondence:** K. Kockum

*Fluids and Barriers of the CNS* 2017, 14(Suppl 1):A36

**Introduction:** Idiopathic normal pressure hydrocephalus (iNPH) is characterized by Hakim’s triad, i.e. impairment of gait and balance, dementia and incontinence. We aimed to describe the relation between Hakim’s triad symptoms and easy accessible radiological markers associated with three cerebral regions hypothesized to contribute to the specific symptomatology i.e. caudate nucleus, hippocampus, and cingulate gyrus.

**Methods:** The material consisted of 168 individuals from the general population (93 females, median age 75, range 66–92 years) with and without signs of iNPH. The triad symptoms were assessed using gait and balance test (TUG), NPH-sensitive neuropsychological tests (Pegboard, Stroop interference, RAVLT), and urinary symptoms (yes/no). The radiological markers Evan’s index (EI), mean size of the temporal horns, and Callosal angle (CA) were measured on CT scans.

**Results:** Relating the three cerebral regions in a multivariate regression analysis to the different symptoms, showed that for TUG, Pegboard and RAVLT, only the size of temporal horns was significantly contributing (p < 0.001 for all three), for Stroop interference CA contributed as well (p = 0.022). Those with urinary symptoms had significantly larger temporal horns (mean = 3.8 mm) than those without (mean = 3.2 mm) (t = −2.38, p = 0.019), whereas no differences were found for EI or CA.

**Conclusions:** More pronounced triad symptoms were related to wider temporal horns on CT. This was not limited to memory impairment. The findings support that wide temporal horns are an important radiological marker of iNPH. However, functional methods are probably needed to link the different symptoms of Hakim’s triad to specific brain regions.

## A37 Prognostic value of radiological findings for mortality in idiopathic normal pressure hydrocephalus

### M. Kojoukhova^1^, A. Sutela^2^, A. M. Koivisto^3,4^, R. Vanninen^2^, J. E. Jääskeläinen^1^, V. Leinonen^1^

#### ^1^Neurosurgery of NeuroCenter, Kuopio University Hospital and University of Eastern Finland, Kuopio, Finland; ^2^Department of Radiology, Kuopio University Hospital and University of Eastern Finland, Kuopio, Finland; ^3^Unit of Neurology, Institute of Clinical Medicine, University of Eastern Finland, Kuopio, Finland; ^4^Neurology of NeuroCenter, Kuopio University Hospital, Kuopio, Finland

##### **Correspondence:** M. Kojoukhova

*Fluids and Barriers of the CNS* 2017, 14(Suppl 1):A37

**Introduction:** Little is known about relationship between radiological markers and mortality in idiopathic normal pressure hydrocephalus (iNPH).

**Methods:** We used data from Kuopio University Hospital’s NPH register of 477 patients with suspected iNPH. A total of 305 (64%) patients underwent shunt surgery and 294 (62%) died during the mean follow-up time of 6.4 years. We used Cox regression model adjusted for BMI, age, gender and imaging method [CT (n = 295) or MRI (n = 182)] to investigate the associations between radiological markers and mortality.

**Results:** White matter changes in brain stem (p < 0.001), periventricular area (p < 0.001) and deep white matter (p = 0.001) were associated with high mortality. Wide temporal horns (p < 0.001) and high Scheltens scores (p < 0.001) were also associated with high mortality. The disproportional Sylvian and supraSylvian subarachnoid spaces (p = 0.026) and decreased superior medial subarachnoid spaces (p = 0.048) were associated with better prognosis. Evans’ index, focally dilated sulci, superior convexity subarachnoid spaces, lateral ventricles, Sylvian fissures, basal cisterns, flow void, callosal angle and modified cella media index were not associated with mortality. In shunted patients the disproportional Sylvian and supraSylvian subarachnoid spaces and decreased superior medial subarachnoid spaces were no longer associated with mortality, while the other associations remained similar.

**Conclusions:** Radiological findings related to Alzheimer’s disease and vascular degeneration were associated with high mortality. However, iNPH related markers were only weakly associated with better survival. Our findings suggest that in the treatment of iNPH patients, we should focus more on vascular degeneration.

## A38 Associations of intracranial pressure with brain biopsy, radiological findings, and shunt surgery outcome in idiopathic normal pressure hydrocephalus

### M. Kojoukhova^1^, K. I. Vanha^1^, M. Timonen^1^, A. M. Koivisto^3,4^, J. Rummukainen^5^, T. Rauramaa^5,6^, R. Vanninen^2^, J. E. Jääskeläinen^1^, A. Sutela^2^, V. Leinonen^1^

#### ^1^Neurosurgery of NeuroCenter, Kuopio University Hospital and University of Eastern Finland, Kuopio, Finland;^ 2^Department of Radiology, Kuopio University Hospital and University of Eastern Finland, Kuopio, Finland;^ 3^Unit of Neurology, Institute of Clinical Medicine, University of Eastern Finland, Kuopio, Finland;^ 4^Neurology of NeuroCenter, Kuopio University Hospital, Kuopio, Finland;^ 5^Department of Pathology, Kuopio University Hospital, Kuopio, Finland;^ 6^Institute of Clinical Medicine-Pathology, University of Eastern Finland, Kuopio, Finland

##### **Correspondence:** M. Kojoukhova

*Fluids and Barriers of the CNS* 2017, 14(Suppl 1):A38

**Introduction:** It remains unclear how intracranial pressure (ICP) measures are associated with brain biopsies and radiological markers. Here, we aim to investigate associations between ICP and radiological findings, brain biopsies, and shunt surgery outcome in patients with suspected idiopathic normal pressure hydrocephalus (iNPH).

**Methods:** We analyzed data from 73 patients admitted with suspected iNPH to Kuopio University Hospital. Of these patients, 71% underwent shunt surgery. The NPH registry included data on clinical and radiological examinations, 24-h intraventricular pressure monitoring, and frontal cortical biopsy.

**Results:** The mean ICP and mean ICP pulse wave amplitude were not associated with the shunt response. Aggregations of Alzheimer’s disease (AD) related proteins (amyloid-β, hyperphosphorylated tau) in frontal cortical biopsies were associated with a poor shunt response (p = 0.014). High mean ICP was associated with Evans’ index (EI; p = 0.025), disproportional Sylvian and supraSylvian subarachnoid spaces (p = 0.014), and focally dilated sulci (p = 0.047). Interestingly, a high pulse wave amplitude was associated with AD-related biopsy findings (p = 0.032), but the mean ICP was not associated with the brain biopsy. The ICP was not associated with medial temporal lobe atrophy, temporal horn widths, or white matter changes.

**Conclusions:** The EI and disproportional Sylvian and Suprasylvian subarachnoid spaces were associated with mean ICP. Their ability, although limited, to predict elevated ICP in 24-h measurements, underlined their value in iNPH diagnostics. Interestingly, our results suggested that elevated pulse wave amplitude might be associated with brain amyloid accumulation.

## A39 Behavioural variant frontotemporal dementia has a potential comorbidity with possible idiopathic normal pressure hydrocephalus

### V. Korhonen^1^, S. Helisalmi^2,3^, E. Solje^2,3^, T. Rauramaa^4^, R. Vanninen^5^, A. M. Remes^2,3^, V. Leinonen^1^

#### ^1^Department of Neurosurgery, University of Eastern Finland and Kuopio University Hospital, Kuopio, Finland; ^2^Institute of Clinical Medicine-Neurology, University of Eastern Finland, Kuopio, Finland; ^3^Department of Neurology, Kuopio University Hospital, Kuopio, Finland; ^4^Department of Pathology, Kuopio University Hospital, Kuopio, Finland; ^5^Department of Radiology, Kuopio University Hospital and University of Eastern Finland, Kuopio, Finland

##### **Correspondence:** V. Korhonen

*Fluids and Barriers of the CNS* 2017, 14(Suppl 1):A39

**Introduction:** Differential diagnosis between idiopathic normal pressure hydrocephalus (iNPH) and other neurodegenerative diseases can be challenging. The *C9ORF72* repeat expansion has been associated with frontotemporal lobar degeneration (FTLD) and the penetrance of the *C9ORF72* mutation is considered complete. We aim to determine the prevalence of the pathological *C9ORF72* repeat expansion in Kuopio University hospital (KUH) NPH-register.

**Methods:** We genotyped the repeat region of *C9ORF72* by using repeat primed polymerase chain reaction (RP-PCR) in a cohort of 551 shunted patients who were all diagnosed with NPH. Frontal cortical biopsy was available for 347 patients. We cross-referenced the *C9ORF72* expansion to neuropathological findings that included beta-amyloid-protein, tau-protein, p62 and TDP-43. All *C9ORF72* expansion positive patients were clinically evaluated after laboratory analysis by a neurologist specialised in FTLD. All results were also read through using amplicon length analysis and positive ones were verified in collaborator’s laboratory.

**Results:** The prevalence of the *C9ORF72* repeat expansion was determined to be 1.1% (6/551). Cortical biopsy was available for five expansion positive patients.

**Conclusions:** Prevalence of the *C9ORF72* expansion in KUH NPH-register is greater than expected and there may be a possible comorbidity between FTLD and iNPH.

## A40 Twenty-three Alzheimer’s disease-related single nucleotide polymorphisms in patients with shunt-responsive INPH: a brief clinical study

### J. Huovinen^1^, S. Helisalmi^2,3^, J. Paananen^2,3,4^, A. M. Koivisto^2,3^, A. M. Remes^2,3^, H. Soininen^2,3^, M. Hiltunen^2,3,4^, M. Kurki^1,5,6,7^, J. E. Jääskeläinen^1^, V. Leinonen^1^

#### ^1^Department of Neurosurgery, Kuopio University Hospital, Institute of Clinical Medicine, University of Eastern Finland, Kuopio, Finland; ^2^Unit of Neurology, Institute of Clinical Medicine, University of Eastern Finland, Kuopio, Finland; ^3^Department of Neurology, Kuopio University Hospital, Kuopio, Finland; ^4^Institute of Biomedicine, University of Eastern Finland, Kuopio, Finland; ^5^Analytical and Translational Genetics Unit, Department of Medicine, Massachusetts General Hospital, Boston, MA, USA; ^6^Program in Medical and Population Genetics, Broad Institute of MIT and Harvard, Cambridge, MA, USA; ^7^Stanley Center for Psychiatric Research, Broad Institute for Harvard and MIT, Cambridge, MA, USA

##### **Correspondence:** V. Leinonen

*Fluids and Barriers of the CNS* 2017, 14(Suppl 1):A40

**Introduction:** Idiopathic normal pressure hydrocephalus (iNPH) is late onset, surgically treated progressive neurodegenerative disease caused by inadequate cerebrospinal fluid (CSF) dynamics and ventriculomegaly. Comorbid Alzheimer’s disease (AD) seems to be more prevalent in patients with iNPH, yet no overexpression of APOE epsilon 4 allele has been discovered in patients with iNPH.

**Materials and methods:** Overall 188 shunt-responsive iNPH-patients and 688 age-matched controls without diagnosed neurodegenerative disease were included into analysis. 23 single-nucleotide polymorphisms (SNP:s) were analysed between groups [FRMD4A (rs7081208_A, rs2446581_A, rs17314229_T), CR1, BIN, CD2AP, CLU, MS4A6A, MS4A4E, PICALM, ABCA7, CD33, INPP5D, HLA_DRB5, NME8, EPHA1, PTK2B, CELF1, SORL1, FERMT2, SLC24A, DSG2, CASS4] between groups. Analyses were performed with SPSS statistics software (version 22.0, SPSS Inc., Chicago, Illinois). Using binary logistic regression analysis, each SNP was analysed with adjustments to age, sex and APOE-genotype.

**Results:** NME8 AG-genotype was more common in iNPH-patients than no demented controls (p = 0.044). With respect to other SNP:s, no other significant differences were discovered between groups.

**Conclusions:** Our findings support iNPH to have independent genetic and pathophysiological mechanisms independent from Alzheimer’s disease. Considering the fact NME8 plays a role in ciliary function and has SNP-related diversity in MRI-volumetry and CSF-biomarkers, the plausible ciliopathic dimension of iNPH as well as further genetic studies are required.

## A41 Intracranial compliance at rest and after infusion in hydrocephalus patients

### A. Lokossou^1^, O. Balédent^1^, S. Garnotel^1^, G. Page^1^, L. Balardy^2^, Z. Czosnyka^3^, P. Payoux^4^, E. A. Schmidt^5,6^

#### ^1^BioFlowImage Laboratory, Department of Medical Image Processing, University Hospital of Picardie Jules Verne, Amiens, France; ^2^Departments of Geriatric, University Hospital of Toulouse, Toulouse, France; ^3^Neurosciences Department, University of Cambridge, Cambridge, UK; ^4^Department of Nuclear Medicine, University Hospital of Toulouse, Toulouse, France; ^5^ UMR 1214–INSERM/UPS–TONIC Toulouse Neuro-Imaging Center, Toulouse, France; ^6^Department of Neurosurgery, University Hospital of Toulouse, Toulouse, France

##### **Correspondence:** A. Lokossou

*Fluids and Barriers of the CNS* 2017, 14(Suppl 1):A41

**Introduction:** Conventionally, intracranial compliance is assessed by considering change in intracranial pressure (ICP) in response to a known volume change injected during the infusion tests (**C_stress**). We propose a new method to assess the physiological intracranial compliance at rest (**C_rest**) without any injection.

**Methods:** 83 subjects (74 ± 7 years) suspected of hydrocephalus underwent a phase contrast magnetic resonance imaging for the quantification of the intracranial liquid volume change (**ΔV**) during cardiac cycle [blood and cerebrospinal fluid (CSF)]. ICP amplitude at rest over the cardiac cycle (**ΔP**) was assessed from ICP monitoring before infusion to calculate **C_rest** = **ΔV/ΔP**. C_stress was calculated as usual by dividing the known injected volume by the pressure amplitude obtained by considering the end and the beginning of the injection. After infusion, resistance to CSF outflow (R_out_) was calculated. Based on their R_out_, the patients were affected in a normal (**Normal_inf**) or a pathological group (**Patho_inf**).

**Results:** In the Normal_inf group, C_stress = 0.93 ± 0.4 ml/mmHg and C_rest = 0.28 ± 0.21 ml/mmHg. In the Patho_inf group, C_stress = 0.62 ± 0.34 ml/mmHg and C_rest = 0.19 ± 0.13 ml/mmHg. For all the patients, C_stress was significantly higher than C_rest. These two compliance parameters were significantly lower in Patho_inf group than in Normal_inf group.

**Conclusions:** We have shown the possibility to assess the physiological intracranial compliance at rest. The values calculated were paradoxically smaller during this short period than during the long period of an infusion test. Nevertheless, because the C_rest values were different between both groups, C_rest could be used as a complementary biomarker to better select the patients who need shunt surgery care.

## A42 CSF fluid dynamics in Chiari malformation: an MRI study of longitudinal impedance

### B. Martin^1^, F. Loth^2^, M. Luciano^3^

#### ^1^Biological Engineering, University of Idaho, Moscow, ID, USA; ^2^Mechanical Engineering, University of Akron, Akron, Ohio, USA; ^3^Neurosurgery, Johns Hopkins University, Baltimore, MA, USA

##### **Correspondence:** M. Luciano

*Fluids and Barriers of the CNS* 2017, 14(Suppl 1):A42

**Introduction:** Chiari malformation 1 is defined as a herniation of the cerebellar tonsils below the foramen magnum and into the spinal canal. Although initially considered a congenital malformation it is increasingly understood as a CSF dynamic disorder where fluid differential pressures and cardiac pulsation may play a role in the pathophysiology. While the degree of descent of the tonsils into the spinal canal has been used as an index of symptom likelihood and candidacy for surgical decompression it has been found to unreliable and fluid dynamic parameters are being investigated.

**Methods:** In this study we investigate the measurement of fluid impedance in the area of the cervicomedullary junction (longitudinal impedance, LI) using quantified phase-contrast MRI imaging in fifteen adult Chiari patients and eight normal controls.

**Results:** Longitudinal Impedance was found to be increased in Chiari patients compared to normal controls and was variably decreased by decompressive surgery. The effect of this increased impedance on pulsatile brain tissue movement will also be discussed.

**Conclusions:** Chiari pathophysiology demonstrates an increased impedance to flow at the cervicomedullary junction that may be related to pathophysiology and symptoms, and that is variably reduced in surgery. The assessment of the cardiac-dynamic fluid-brain interaction in Chiari malformation may provide insights into the pathophysiology, symptom generation, and appropriate surgical treatment.

## A43 The disease state index outperforms cerebrospinal fluid tap test in shunt surgery outcome prediction

### A. J. Luikku^1,2^, A. Hall^1^, O. Nerg^1,3^, A. M. Koivisto^1,3^, M. Hiltunen^1,3,4^, S. Helisalmi^1^, A. Junkkari^2^, S. K. Herukka^1,3^, A. Sutela^5^, M. Kojoukhova ^2,5^, J. Mattila^6,7^, J. Lötjönen^6,7^, J. Rummukainen^8^, I. Alafuzoff^1,9,10^, J. E. Jääskeläinen^2^, A. M. Remes^1,3^, H. Soininen^1,3^, V. Leinonen^2^

#### ^1^Institute of Clinical Medicine-Neurology, University of Eastern Finland, Kuopio, Finland; ^2^Neurosurgery of NeuroCenter, Kuopio University Hospital, Kuopio, Finland; ^3^Neurology of NeuroCenter, Kuopio University Hospital, Kuopio, Finland; ^4^Institute of Biomedicine, University of Eastern Finland, Kuopio, Finland; ^5^Department of Radiology, Kuopio University Hospital, Kuopio, Finland; ^6^VTT Technical Research Centre of Finland, Tampere, Finland; ^7^Combinostics Ltd, Tampere, Finland; ^8^Department of Pathology, Kuopio University Hospital, Kuopio, Finland; ^9^Rudbeck Laboratory, Department of Immunology, Genetics and Pathology, Uppsala University, Uppsala, Sweden; ^10^Department of Pathology and Cytology, Uppsala University Hospital, Uppsala, Sweden

##### **Correspondence:** A. J. Luikku

*Fluids and Barriers of the CNS* 2017, 14(Suppl 1):A43

**Introduction:** Optimal selection of idiopathic normal pressure hydrocephalus (iNPH) patients for shunt surgery is challenging. Cerebrospinal fluid tap test (CSF-TT) is often used as a prognostic test for shunt surgery outcome evaluation, but its negative predictive value (NPV) is poor. Disease State Index (DSI) is a statistical method that merges multimodal data to assist clinical decision-making. In this study we predict shunt surgery response measured with iNPH Grading Scale (iNPH-GS) for patients with iNPH.

**Methods:** 93 patients (42 shunt responders and 51 non-responders) for 3-month outcome and 97 patients (44 shunt responders and 53 non-responders) for 1-year outcome were analyzed with the DSI. iNPH-GS score and subscores, status of first and present symptoms, previous cerebrovascular disease and mean walking speed after CSF-TT were significant in our analysis. Statistical significance between groups was not observed with MMSE, ADCS-ADL-questionnaire, CT/MRI imaging, APOE-genotyping and CSF AD-biomarkers. Prognostic power for both DSI and CSF-TT were evaluated with Receiver Operating Characteristic (ROC) Curve analysis.

**Results:** DSI was able to predict shunt surgery outcome effectively both for 3 months (ACC 65.9%, SEN 66.8%, SPE 65.1%, PPV 61.2%, NPV 70.5%) and 1 year (ACC 68.6%, SEN 66.6%, SPE 70.2%, PPV 65.0%, NPV 71.7%). CSF-TT was not useful for outcome prediction neither for 3 months (ACC 47.3%, SEN 50.0%, SPE 45.1%, PPV 42.9%, NPV 52.3%) nor 1 year (ACC 47.4%, SEN 47.7%, SPE 47.2%, PPV 42.9%, NPV 52.1%). Merging CSF-TT and DSI did not improve predictive value.

**Conclusions:** DSI can be used reliably to predict shunt surgery outcome, even for 1 year.

## A44 Relationship between intra-abdominal pressure and outcome in idiopathic intracranial hypertension

### S. Matloob, C. Craven, S. Thompson, I. Baudracco, A. K. Toma, L. D. Watkins

#### Victor Horsley Department of Neurosurgery, National Hospital for Neurology and Neurosurgery, London, UK

##### **Correspondence:** S. Matloob

*Fluids and Barriers of the CNS* 2017, 14(Suppl 1):A44

**Introduction:** IIH is more common in patients with a raised BMI. Increased intra-peritoneal pressure is likely to affect the functioning of CSF shunts. We aimed to investigate the relationship between intra-abdominal pressure and outcomes in patients with IIH.

Idiopathic intracranial hypertension (IIH) is a difficult to treat condition. In a subset of patients, this condition results from venous stenosis. Stenosis can be primary or secondary to another cause. One theory is increased abdominal mass compresses the IVC giving rise to increased venous pressures resulting in a similar picture to IIH. We aimed to investigate the relationship between BMI and outcomes in patients with IIH.

**Methods:** Case notes and radiological reports were reviewed. BMI for each patient was recorded as well as what interventions each patient had. Inferior vena cava pressure (IVCP) measured during venography was recorded. The outcome in clinic was also recorded.

**Results:** We identified 27 patients who had undergone venography as part of their assessment for IIH. IVCP ranged from 3 to 19 mmHg (10.7 ± 5.04). 21/27 patients had IVCP >8 mmHg. BMI ranged from 25.9 to 63.4. Mean follow up was 9.4 months. Of these 27 patients 15 had undergone CSF drainage into the peritoneum. 60% of these patients had a BMI >30. We found a positive correlation between BMI and need to re-operate. These patients were advised to have bariatric surgery or pleural shunts.

**Conclusions:** Outcome was less likely to resolve symptoms in patients with an increased BMI. For patients with CSF drainage into the peritoneum the need for re-intervention was higher and the outcome in clinic was worse. Therefore patients with a raised BMI and evidence of IIH should be considered for atrial or pleural shunt or be considered for bariatric intervention.

## A45 Venous sinus stenting for idiopathic intracranial hypertension: 2 year follow up study of 40 patients

### S. Matloob, C. Craven, S. Thompson, I. Baudracco, A. K. Toma, L. D. Watkins

#### Victor Horsley Department of Neurosurgery, National Hospital for Neurology and Neurosurgery, London, UK

##### **Correspondence:** S. Matloob

*Fluids and Barriers of the CNS* 2017, 14(Suppl 1):A45

**Introduction:** Venous sinus stenting is increasingly being used as a treatment option in patients with idiopathic intracranial hypertension. Venous hypertension is frequently observed in patients with IIH, and the pathophysiology behind this remains poorly understood. Pressure gradients across focal points of stenosis in the venous sinuses have been demonstrated and this pressure gradient has been shown to be obliterated once a stent is inserted. Furthermore we have been able to demonstrate a significant reduction in the ICP within 24 h after stenting. We aim to present the follow up findings for these difficult to treat patients from our unit.

**Methods:** A case note review was carried out for all patients with idiopathic intracranial hypertension that had undergone venous sinus stenting. Length of follow up was recorded and clinical outcome as well as ICP was noted.

**Results:** 40 patients who had undergone venous sinus stenting were identified. Mean length of follow up was 24.1 months (range 8–60 months). 1 patient had a clinically silent intracerebral bleed during the stent insertion. 95% (38/40) of our patient cohort were female. On follow up venography 97.5% (39/40) of our patients demonstrated a significant reduction in their pressure gradient. Mean intracranial pressure was recorded following stenting in 27 patients, and of these 27 patients a significant reduction in ICP was recorded in 23 patients. Clinical improvement was recorded in 42.5% (17/40) of patients.

**Conclusions:** Venous sinus stenting is associated with a significant reduction in intracranial pressure and is effective in obliterating a pressure gradient across the venous sinus. This however does not correlate with clinical improvement, particularly with regards to improving symptoms of headache.

## A46 Potential microRNA biomarkers for diagnosis of idiopathic normal pressure hydrocephalus

### I. Jurjević^1,2^, M. Miyajima^1^, M. Nakajima^1^

#### ^1^Department of Neurosurgery, Graduate School of Medicine, Juntendo University, Tokyo, Japan; ^2^Department of Pharmacology and Department of Neurology, University of Zagreb School of Medicine, Zagreb, Croatia

##### **Correspondence:** M. Miyajima

*Fluids and Barriers of the CNS* 2017, 14(Suppl 1):A46

**Introduction:** Patients presenting with idiopathic normal pressure hydrocephalus (iNPH) triad often show some additional symptoms from the Parkinsonian spectrum (PS) including progressive supranuclear palsy (PSP), or symptoms typical for Alzheimer’s disease (AD). Making the correct initial diagnosis is challenging, but it also strongly influences the long-term outcome of shunting procedures. The aim of this study was to find potential cerebrospinal fluid (CSF) microRNA biomarkers to distinguish patients with iNPH from PS and AD.

**Methods:** Records of 55 patients with iNPH that were treated between the year 2011 and 2014 were retrospectively reviewed. CSF samples were obtained from patients clinically diagnosed with definite iNPH (n = 21), possible iNPH with PS (n = 18), and possible iNPH with AD (n = 16). A two-step qRT-PCR analysis of the CSF samples was done to detect miRNAs that were differently expressed in the definite iNPH group.

**Results:** From 1008 miRNAs, miR-1280 and miR-4274 showed most promising results, both distinguishing definite iNPH versus possible iNPH with PS (AUC = 0.997 and 0.913), and miR-1280 distinguishing definite iNPH versus possible iNPH with AD (AUC = 0.949) with high accuracy. Furthermore, the combination of these microRNA biomarkers and p-Tau clearly separated iNPH from PS and AD patients.

**Conclusions:** Potential microRNA biomarkers were identified in CSF of definite iNPH patients that could differentiate them from patients presenting with symptoms that are overlapping with other neurodegenerative disorders. Further investigations on larger number of patients would be useful to confirm these preliminary results.

## A47 Antithrombotic drugs or neurological comorbidities might not be a contraindication for shunt surgery on patients with idiopathic normal pressure hydrocephalus

### H. Murai, T. Shin, D. Kawaguchi

#### Department of Neurosurgery, Graduate School of Medicine, Chiba University Chiba, Japan

##### **Correspondence:** H. Murai

*Fluids and Barriers of the CNS* 2017, 14(Suppl 1):A47

**Introduction:** Idiopathic normal pressure hydrocephalus (iNPH) is a disease of the elderly, and there is a possibility that a number of complications coexist. Shunt surgery if it is properly indicated and performed improves the patient’s QOL, but it might increase the risk of subdural hematoma (SDH). So we examined the effects of antithrombotic drugs or neurological comorbidities on the outcome.

**Methods:** From July 2002 to May 2015, 290 cases of iNPH were diagnosed. In the 171 patients who underwent shunt surgery, comorbidities, preoperative cognitive function and the effects of antithrombotic drugs on SDH were examined.

**Results:** Of the 171 patients subjected to surgery, neurological comorbidities with dementia coexisted in 45 patients (26%), oral antithrombotic drugs were taken in 59 cases (34%). A median observation period was 3 years and 10 months. Chronic subdural hematoma was observed in 29 patients (17%, 4% per year), with non-surgical treatments the surgery was needed in only 7 patients (4.1%, 0.96% per year). Antithrombotic agents have the trend to increase the incidence, but it was not significant. There was no correlation between the outcome and preoperative cognitive function. Even if there is coexistence of other neurological disorders, most cases had some better period before deterioration.

**Conclusions:** (1) In case of coexistence with dementia, we recommend doing tap test even if the MRI image is typical and informing and negotiating well with the patients and their family. (2) The use of antithrombotic drugs did not appear to be a contraindication for shunt surgery.

## A48 Comparison of lumboperitoneal shunt systems using small lumen abdominal catheter versus gravitational add-on valve in idiopathic normal pressure hydrocephalus

### M. Nakajima, M. Miyajima, C. Akiba, I. Ogino, K. Karagiozov, H. Arai

#### Department of Neurosurgery, Juntendo University School of Medicine, Tokyo, Japan

##### **Correspondence:** M. Nakajima

*Fluids and Barriers of the CNS* 2017, 14(Suppl 1):A48

**Introduction:** Cerebrospinal fluid (CSF) shunt also has a role of improving clearance of CSF in addition to the role of adjusting intracranial pressure. Neurotoxic proteins in CSF are removed from the brain by shunt treatment. For lumboperitoneal shunt (LPS) that is used for treatment of iNPH, complications arising from CSF over-drainage (OD) are perceived as problematic.

The objective of treatment is to avoid complications caused by OD, while maintaining the amount of CSF drained. For that it is necessary to create a shunt system that can avoid OD and also safely facilitate removal of neurotoxic proteins such as Aβ42. We aimed to establish whether LPS by a shunt system that has gravitational add-on valves (GV) installed in tandem with programmable pressure valve could reduce complications arising from OD due to iNPH and improve the condition of patients.

**Methods:** The peritoneal catheter has a small (0.7 mm) inner lumen (SL) to preserve distal shunt resistance. GV, enables the surgeon to use different opening pressure for supine and standing positions, managing over drainage complications and patient discomfort.

We compared two settings of Strata NSC valve shunt systems: one with attached GV and one with SL, under different performance levels.

We analyzed 62 cases of iNPH patients (average age of 75.0 years) to whom LPS was implanted using GV + programmable pressure valves in tandem from March 2013 through June 2015, and 54 cases (74.7 years) of treatment of iNPH from July 2010 through April 2012. By shunt systems with programmable pressure valves + SL with regard to OD complications 1 year after commencement of treatment, evaluating mRS, Japan NPH grading scale score, MMSE and CSF biomarkers; amyloid beta (Aβ) 42, p-tau.

**Results:** As for complications due to OD, on the other hand, the rate of postoperative headache was 13.2% and that of chronic subdural hematoma that required surgery was 2.2%, showing a decreasing trend. mRS scores improved from 2.8 to 2.2, total JNPHGS decreased from 5.9 to 4.5. MMSE scores improved from 21.9 to 24.8. Aβ42, p-tau concentration were changed from 483 to 480 (pg/ml), 31.3 to 71.9 (pg/ml) respectively.

**Conclusions:** It was found that the complications of low CSF pressure syndrome peculiar to LP shunt tended to be decreased by the use of the add-on valve system. In the case where GV was used in tandem, the effect of insufficient removal of CSF was assumed.

## A49 Effect of low-level lasertherapy in patients with pain among ventriculoperitoneal catheter

### R. C. Reis^1^, M. J. Teixeira^1^, C. G. Valêncio^1^, D. da Vigua^1^, L. Almeida-Lopes^2^, M. W. Mancini^2^, F. C. G. Pinto^1^

#### ^1^Group of Cerebral Hydrodynamics, Division of Functional Neurosurgery, Institute of Psychiatry, Hospital das Clínicas, University of São Paulo, São Paulo, Brazil; ^2^Núcleo de Pesquisa e Ensino de Fototerapia nas Ciências da Saúde (NUPEN), São Carlos, Brazil

##### **Correspondence:** F. C. G. Pinto

*Fluids and Barriers of the CNS* 2017, 14(Suppl 1):A49

**Introduction:** Pain around the catheter is a common complaint in patients with ventriculoperitoneal shunt. Low-level lasertherapy (LLLT) has been applied in the treatment of different types of pain. We sought to evaluate LLLT efficiency in treating ventriculoperitoneal catheter pain.

**Methods:** Randomized, placebo-controlled, double-blind study with patients treated in the clinic of a tertiary referral hospital complaining of pain around catheter. They were randomized in two groups: one treated with LLLT (infrared diode laser, Therapy XT EC: 808 nm) and the other received sham-laser. Applications were done twice a week, through 4 weeks. Patients evaluated pain intensity before first session of lasertherapy and after 2, 4 and 8 weeks of treatment, using pain visual analog scale (VAS) and McGill questionnaire.

**Results:** Until now, 2 patients were randomized to LLLT group and 2 to sham-laser group. Patients submitted to LLLT had an initial score of 7 in VAS, turning to a mean score of 3 in 4 weeks. When submitted to McGuill questionnaire, pain score also decreased from 24 to 5. One month after treatment, though, both scores increased: mean of 6 in VAS and 20 in McGuill. Patients treated with sham-laser did not get better during treatment: VAS score decreased from 8 to 7 and McGuill decreased from 19 to 18. There were no side effects of LLLT in this study.

**Conclusions:** Our results showed important analgesic action of LLLT in patients complaining of ventriculoperitoneal catheter pain, without side effects. This effect seems to be temporary and dependent of multiple applications.

## A50 Diagnosis and treatment of normal pressure hydrocephalus by a multidisciplinary team

### R. H. Maykot, G. Calia, J. Tornai, SSS Silvestre, G. Mendes, V. Sousa, B. Bezerra, P. Dutra, P. Modesto, M. F. Oliveira, R. C. Reis, C. E. Petitto, F. C. Pinto

#### Group of Cerebral Hydrodynamics, Division of Functional Neurosurgery, Institute of Psychiatry, Hospital das Clínicas, University of São Paulo, Brazil

##### **Correspondence:** F. C. Pinto

*Fluids and Barriers of the CNS* 2017, 14(Suppl 1):A50

**Introduction:** Normal pressure hydrocephalus (NPH) is characterized by gait apraxia, urinary incontinence and several degrees of dementia. Usual diagnosis is based on classic triad and neurological imaging and advocated treatment needs a shunt placement. Nevertheless, there are few reports of a full multidisciplinary team evaluation, in order to maximize diagnosis and outcomes, including neuropsychologists, nutritionist, music therapists and physical therapists

**Methods:** From 2014 to 2015, a pilot evaluation of 25 patients with NPH was performed, according to specific music therapy evaluation, neuropsychological tests and physical therapy in addition to neurological and neurosurgical approach. Patients were evaluated before and after surgery of ventriculoperitoneal shunt, with a follow-up of 1 year. We evaluated pre and post operative body mass index, Mini-Mental State Examination, Timed Up and Go and a music therapy assessing tool.

**Results:** The evaluation in a multidisciplinary approach increased sensibility for diagnosing NPH. We did not perform VPS only with clinical and radiological patterns, but also with positive results in multidisciplinary evaluation. Additionally, the presence of neuropsychologist improved cognitive rehabilitation and motor rehabilitation improved by physical therapist.

**Conclusions:** A multidisciplinary team is able to increase sensibility in diagnosing and following patients with NPH before and after surgery, detecting subtle alterations and allowing maximal functional rehabilitation.

## A51 Results of vision study (venous intervention versus shunting in IIH for optic disc swelling) trial patient questionnaire

### H. Pulhorn^1^, A. Chandran^2^, C. McMahon^1^

#### ^1^Department of Neurosurgery, The Walton Centre, Liverpool, UK; ^2^Department of Neuroradiology, The Walton Centre, Liverpool, UK

##### **Correspondence:** H. Pulhorn

*Fluids and Barriers of the CNS* 2017, 14(Suppl 1):A51

**Introduction:** Headaches, visual problems and tinnitus are symptoms of idiopathic intracranial hypertension (IIH) which resolve with reduction of CSF pressure. Impaired cranial venous outflow has been implicated in the pathogenesis and there is evidence of good treatment results in IIH using venous sinus stenting. We are currently initiating a multi-centre randomised controlled trial, the VISION study (Venous Intervention versus Shunting in IIH for Optic Disc Swelling) comparing radiological (venous sinus stenting) to surgical intervention (CSF shunting).As part of the preparation for VISION we made a basic questionnaire available to members of the website IIH UK (http://www.iih.org.uk).

**Methods:** 10-point questionnaire pertaining to IIH diagnosis, symptoms and management using http://www.surveymonkey.com.

**Results:** 250 questionnaires were returned. 95.6% of respondents were female, mostly ≤40 years of age. 70% were diagnosed in the last 5 years, but only 35% were diagnosed less than a year after onset of symptoms. 59.4% of patients had not undergone any radiological/surgical intervention, 34.9% had had CSF diversion, 3.6% venous stenting and 2.0% had stent plus shunt. 16.8% indicated their lives were most affected by tinnitus and 18.1% by visual problems, but 49.6% said they were most affected by their headaches. 81% of patients indicated they would be happy to participate in a randomised trial comparing the two treatment options of venous stenting and CSF shunting.

**Conclusions:** IIH patients want to be actively involved in their treatment and are favourably disposed towards clinical research. Variation exists in treatment modalities offered. There are individual differences regarding impact of symptoms.

## A52 Sleep apnea and leptin resistance in idiopathic intracranial hypertension syndrome

### A. S. Rao, M. Jumaly, D. Solomon, A. Moghekar

#### The Johns Hopkins Hospital, Baltimore, MD, USA

##### **Correspondence:** A. S. Rao

*Fluids and Barriers of the CNS* 2017, 14(Suppl 1):A52

**Introduction:** Obesity is a risk factor for both idiopathic intracranial hypertension syndrome (IIHS) and sleep apnea (SA). Studies have demonstrated the relationship between elevations in intracerebral pressure and sleep apnea as well as shown a relationship between papilledema and OSA. In our sample 39% of patients with IIHS were shown to have SA. Thus far, there are no human studies on neurochemical factor that may play a role in the susceptibility of this population to sleep disordered breathing. Current literature has shown low leptin levels in obese patients with SA. In addition leptin is well known for its adipogenic properties. Animal studies have demonstrated a role for the adipocytokines especially Leptin which is deficient in the cerebrospinal fluid (CSF) leptin in obese animals, a phenomenon is known as of “leptin resistance”. However, this has not been tested in patients with IIHS. Our hypothesis is that patients with IIHS are more susceptible to OSA due to obesity and/or leptin resistance. Through this study we plan to explore further the anthropometric characteristics that make subjects with IIHS more susceptible to sleep disordered breathing. We also plan to analyze the CSF from these subjects for evidence of leptin resistance as well as other possible neurohumoral factors which may play a role in the pathophysiology. Objectives: (1) To identify the underlying clinical characteristics of Obstructive Sleep Apnea (OSA) in patients diagnosed with Idiopathic intracranial hypertension syndrome (IIHS). (2) To characterize the relationship between obesity and leptin levels in patients with both IIHS and OSA.

**Methods:** Adults ≥18 years of age in The Johns Hopkins center for CSF disorders with a confirmed diagnosis of IIHS who provided informed consent were selected for the study. These patients underwent an evaluation that included a history (including a sleep and headache questionnaire) and physical exam, lumbar puncture to measure opening pressure and a detailed examination for visual abnormalities. Neuroimaging was obtained as part of the diagnostic workup. For our project we will include a detailed sleep history administered by the Sleep Fellow (including The Epworth sleepiness score and the Hopkins sleep survey) and examination relevant to sleep disordered breathing (including BMI, neck circumference and examination of the oropharynx) to identify those with SA risk factors.

Patients who were identified as high probability of having sleep apnea underwent an overnight polysomnogram. The sleep studies were scored using standard AASM scoring criteria.

CSF and blood which is obtained as part of routine care was tested for leptin levels in all subjects.

*Independent variables* Opening pressure, CSF leptin, plasma leptin, apnea-hypopnea index, oxygen desaturations, arousals, and carbon dioxide recordings where available.

*Covariates* We recorded demographic and anthropometric measurements, medical comorbidities and medications.

**Results:** Data from a total of 18 patients was collected. Higher opening pressures were found in subjects with higher BMI (*p* = 0.03) and SA (*p* = 0.05). Leptin concentrations in CSF were not correlated to the plasma level (*r* = 0.5; *p* = 0.216). CSF leptin levels were not correlated to body mass index (*r* = 0.14; *p* = 0.567). Opening pressure did not correlate with CSF leptin levels (*r* = 0.96*; p* = 0.732)

**Conclusions:** Obesity is a risk factor for both PTC and OSA and is characterised by leptin resistance due to poor penetration through the blood–brain barrier. Leptin has been shown to be protective against OSA. Our pilot data demonstrates that in subjects with IIHS those with higher BMI and SA had higher opening pressures. Our analysis of leptin levels in subjects with IIHS and SA did not demonstrate a correlation between CSF and serum leptin as is seen in obesity and SA, i.e. “leptin resistance”. There was also no correlation with BMI which was a surprising finding. This suggests that the neurochemical pathways associated with leptin in IIHS are unique and distinct from its role in adiposity.

## A53 A comparison of four etiologic subtypes of adult hydrocephalus from the AHCRN patient registry

### N. Relkin^1^, M. Hamilton^2^, H. Katzen^3^, M. Williams^4^, T. Bach^5^, S. Zuspan^5^, R. Holubkov^5^

#### ^1^Department of Neurology, Weill Cornell Medical College, New York, NY, USA; ^2^Department of Neurosurgery, University of Calgary, Alberta, Canada; ^3^Department of Neurology, University of Miami, Miami, FL, USA; ^4^Department of Neurosurgery, Washington University, Seattle, WA, USA; ^5^Utah Data Collection Center (DCC), University of Utah, Salt Lake City, UT, USA

##### **Correspondence:** N. Relkin

*Fluids and Barriers of the CNS* 2017, 14(Suppl 1):A53

**Introduction:** This is the first multicenter observational study to compare four different etiologic subtypes of hydrocephalus in adults: idiopathic (iNPH), acquired (aNPH), transitional (t-Hydro) and congenital (c-Hydro). This interim analysis was triggered by accrual of 200 subjects from five sites in the US and Canada in the Adult Hydrocephalus Clinical Research Network (AHCRN).

**Methods:** Informed consent was obtained under IRB-approved protocols from subjects ≥18 years who met pre-specified clinical and imaging criteria. This study examined baseline demographics, co-morbidities and selected clinical measures. Analyses included descriptive statistics and t-tests to evaluate the significance of group differences.

**Results:** Subjects ranged in age from 18 to 88 years of age with a slight predominance of males. The iNPH group was the oldest and tHydro the youngest. Percentages of subjects with the respective subtypes of hydrocephalus varied significantly across sites. All t-Hydro subjects were treated by shunt or ETV, whereas the majority of others were untreated. Hypertension was most common in iNPH and relatively rare in t-Hydro and c-Hydro. Epilepsy was over-represented in tHydro. Increased frequencies of hypertension, diabetes, coronary artery disease, spinal stenosis, obesity and sleep apnea were observed in iNPH. Gait, urinary and cognitive measures showed the greatest impairment in iNPH.

**Conclusions:** This pilot study highlights important group differences exist among adults with four hydrocephalus etiologies. Differences in rates of enrollment by etiologic subtype across sites may have contributed ascertainment bias. Age and treatment status are possible co-variates. A possible association between sleep apnea and iNPH has not been widely reported and deserves further study.

## A54 Variations in healthcare expenditures of Medicare beneficiaries with hydrocephalus over a decade: 1999 versus 2008

### A. Rigamonti^1^, J. Lu^2^, J. Robison^2^, A. Adam^2^, G. Clemens^3^, P. Sharkey^4^, K. Carson^3^, A. Sanyal^3^, T. Vivas-Buitrago^2^, I. Jusue-Torres^2^, J. Hoffberger^2^, E. Sankey^2^, D. Rigamonti^2^

#### ^1^Cornell University, Ithaca, NY USA; ^2^Department of Neurosurgery, Johns Hopkins University, Baltimore, MD, USA; ^3^Bloomberg School of Public Health, Johns Hopkins University, Baltimore, MD, USA; ^4^School of Business, Loyola University Maryland, Baltimore, MD, USA

##### **Correspondence:** A. Rigamonti

*Fluids and Barriers of the CNS* 2017, 14(Suppl 1):A54

**Introduction:** In 2007 Williams et al. published an analysis of total Medicare costs for patients with a diagnosis of Hydrocephalus using Medicare data from 1999. Costs significantly changed over the last decade. This study’s goal was to determine whether Hydrocephalus diagnosis prevalence and surgical treatment (including associated costs) of Medicare patients have changed over time. The impact of costs averted from Hydrocephalus due to these interventions was reviewed.

**Methods:** This retrospective cost analysis, mirroring Williams et al., 2007 study was performed using the standard analysis files of paid claims for beneficiaries enrolled in both part A (inpatient) and part B (outpatient) of the Medicare program for 2008. Some outcome measures were Hydrocephalus prevalence, 5-year total direct medical payment and costs by specific service-types.

**Results:** In 2008 alone, 2378 patients, 0.2% of the Medicare beneficiaries were diagnosed with Hydrocephalus (a 1.65-fold increase from 0.12% in 1999). Of these, 637 patients, 26.8%, underwent shunt surgery (an increase from the 25.1% in 1999). 2008 data suggests on average shunted patients spend $10,256 ($2051/year) more over 5 years than patients without shunts. This is dramatically different than 1999 data, which showed patients saved on average $25,477 ($5095/year) over a 5-year span. The majority of this change comes from hospital costs, which shifted from $18,565 cheaper to $10,737 more expensive for shunted patients over 5 years.

**Conclusions:** From 1999 to 2008 the rate of Hydrocephalus diagnosis increased 1.65-fold. Besides the increase of confirmed cases, both surgical intervention and subsequent hospitalization costs have significantly increased.

## A55 Assessing the burden of care of the elderly in the Kingdom of Saudi Arabia

### K. Rigamonti^1^, J. Robison^1^, J. Lu^1^, A. Adam^1^, A. Sattar^2^, O. Omoush^2^, S. Naqvi^3^, T. Vivas Buitrago^1^, D. Rigamonti^1^

#### ^1^Department of Neurosurgery, Johns Hopkins University, Baltimore, MD, USA; ^2^Primary Care, Johns Hopkins Aramco Healthcare, Ras Tanura, Saudi Arabia; ^3^Primary Care, Johns Hopkins Aramco Healthcare, Abqaiq, Saudi Arabia

##### **Correspondence:** K. Rigamonti

*Fluids and Barriers of the CNS* 2017, 14(Suppl 1):A55

**Summary:** Stress of caring for an elderly person can have a negative impact on a caregiver’s health and well-being. Lack of functional independence in the elderly has been shown to increase caregiver’s burden, both in the quantity of time and resources needed as well as changes in personal health relationships of the caregiver in charge. But establishing associations in caregiver’s burden across different culture and regions in the world is unclear and less studied. Given the lack of knowledge on the caregiver-patient structures seen within Johns Hopkins Aramco Healthcare (JHAH), the primary aims of this exploratory study are to (1) define caregiver-patient demographics for the region and (2) identify the relationship between aspects of caring context and caregiver health as measured by quality of life. This study involves prospective data collection of geriatric patients seen at JHAH who will be assessed on their ability to carry out their activities of daily living by administering the Barthel Index. Eligible patients and their primary caregivers will be asked to participate in the voluntary survey. The caregivers will be assessed on their quality of life (SF-36) and burden from caregiving role (Zarit burden questionnaire). The patients will be assessed on their quality of life (SF-36). This study clarifies the type of caregiver and caregiver duties specific to JHAH, the effect of the burden on the caregiver and ways to improve caregiver well-being.

## A56 Lumbar punctures may predict the quantitative gait improvement expected post-shunting in patients with iNPH

### J. Lu, J. Robison, A. Adam, T. Vivas-Buitrago, A. Hung, D. Rigamonti

#### Department of Neurosurgery, Johns Hopkins University, Baltimore, MD, USA

##### **Correspondence:** J. Robison

*Fluids and Barriers of the CNS* 2017, 14(Suppl 1):A56

**Introduction:** Lumbar punctures (LP) are often used as a diagnostic measure in patients with idiopathic normal pressure hydrocephalus (iNPH) as they mimic the effects of ventricular shunting. Gait improvement after an LP is used to determine which patients could benefit from undergoing surgical treatment for iNPH. In this abstract, we seek to correlate the quantitative improvements in post-LP gait assessment with the improvements in post-shunt gait assessments.

**Methods:** All patient records of shunt procedures by the senior author at Johns Hopkins Medical Institution, from 1993 to 2014, were reviewed (n = 150). All patients completed gait evaluation at baseline, post-LP, and again 6 months post shunt surgery. Clinical outcomes measured were Tinetti Gait and Balance Scale and Timed Up and Go test (TUG). Wilcoxon signed rank tests were performed using STATA 13.0.

**Results:** The mean improvement at post-LP and 6-month from baseline TUG was 4.6 (2.0–7.2) and 5.8 (0–11.7) s respectively and 2.9 (2.3–3.6) and 4.2 (2.6–5.8) points respectively on baseline Tinetti. While both tests show significant statistical improvement from baseline at post-LP and 6 months (p value <0.001), only the TUG showed statistical improvement in the post-LP to 6-month period (p value <0.001).

**Conclusions:** Patients showed as much improvement in all gait parameters at 6 months as they did after the diagnostic LP. For the TUG test, patients can actually expect to see even more improvement post-surgery than they saw after the initial LP.

## A57 Dopaminergic loss in patients suspect of hydrocephalus

### E. Schmidt^1,5^, F. Ory-Magne^2,5^, L. Balardy^3^, P. Gantet^4^, P. Payoux^4,5^

#### ^1^Department of Neurosurgery, University Hospital Toulouse, Toulouse, France; ^2^Department of Neurology, University Hospital Toulouse, Toulouse, France; ^3^Department of Geriatry, University Hospital Toulouse, Toulouse, France; ^4^Department of Nuclear Medicine, University Hospital Toulouse, Toulouse, France; ^5^INSER TONIC 1014, Toulouse Neuroimaging Center, Toulouse, France

##### **Correspondence:** E. Schmidt

*Fluids and Barriers of the CNS* 2017, 14(Suppl 1):A57

**Introduction:** Dementia with Lewy bodies (DLB) is the third most common cause of dementia after Alzheimer’s disease and vascular dementia. Diagnosis of probable DLB can be made by fluctuating mental status, visual hallucinations, and mild Parkinsonism with hypokinetic gait. DLB is associated with a significant loss of dopaminergic cell. This loss can be confirmed in vivo with DaTcan, a brain imaging of the dopamine transporter. Hydrocephalus is often associated with a Parkinsonism. The aim of this work is to assess the dopaminergic denervation in a group of patients suspected of NPH.

**Methods:** A prospective cohort of 86 patients suspected of NPH has been included. For every patient, a lumbar infusion study has been performed with calculation of hydrodynamic parameters. DaTscan has been performed with image analysis by DaTsoft3D software for quantification of the dopaminergic neurons activity. We quantified the left and right activities of the caudate nucleus and putamen. We also calculate the putaminal index (PI = min of putamen − 0.5 × |right putamen − left putamen|. This index allows below two indicates a dopaminergic cell loss vs. healthy controls.

**Results:** In our group, the mean activities were: right caudate: 3.54 ± 0.94, right putamen: 2.48 ± 0.89, left caudate: 3.45 ± 0.98, left caudate: 2.38 ± 0.89 and putaminal index: 1.88 ± 0.91 that indicates a clear dopaminergic denervation. There was no correlation between the hydrodynamic parameters and the dopaminergic activity.

**Conclusions:** We demonstrate a dopaminergic denervation in our patients suspected of NPH but did not identify in a preliminary analysis any subgroups of patients.

## A58 Intracranial venous pulsatility is reduced after transverse sinus stenosis

### A. Guenego^1,2^, A. C. Januel^1^, P. Tall^1^, N. Fabre^3^, L. Mahieu^4^, Z. Czosnyka^5^, C. Cognard^1^, E. A. Schmidt^2^

#### ^1^Department of Neuroradiology, University Hospital Toulouse, Toulouse, France; ^2^Department of Neurosurgery, University Hospital Toulouse, Toulouse, France; ^3^Department of Neurology, University Hospital Toulouse, Toulouse, France; ^4^Department of Ophtalmology, University Hospital Toulouse, Toulouse, France; ^5^Brain Physics Lab, Academic Neurosurgery, University of Cambridge, Cambridge, UK

##### **Correspondence:** E. A. Schmidt

*Fluids and Barriers of the CNS* 2017, 14(Suppl 1):A58

**Introduction:** Transverse sinus stenosis is seen in the majority of patients with idiopathic intra-cranial hypertension (IIH). In case of a significant pressure gradient, transverse sinus is stented to reduce cerebral venous pressure, improve CSF resorption, reduce ICP and papilledema. However the pathogenesis of sinus stenosis and its effect on the cerebral venous system remains controversial. We hypothesize that transverse sinus stenosis stenting modifies the dynamic component of venous pressure.

**Methods:** Ten IIH patients were prospectively enrolled. Under general anesthesia, a microcatheter was navigated into cerebral veins and sinuses. Intra sinus pressure was measured and recorded upstream and downstream from the stenosis with a pressure transducer connected to the micro catheter. A stent was placed if a significant pressure gradient (>10 mmHg) was found across the stenosis. Finally pressure was measured upstream and downstream from the stented stenosis. Off line mean venous pressure (VP) was calculated and waveform analyses were performed to extract fundamental harmonic A1 (heart-rate), second harmonic A2 (2 * heart-rate), and respiratory component (Resp). Parameters are presented before and after stenting (mean ± SD) upstream and downstream the stenosis with p value (Kruskal–Wallis test).

**Results:** Stenting significantly reduces mean venous pressure upstream from the stenosis, which is expected. Stenting also significantly reduces fundamental harmonic A1 upstream with a trend in A1 decrease downstream the stenosis (Table 1).

**Table 1:** Effect of venous stenting on venous pressure, venous pulsatility and respiratory component

	Before stent	After stent	% change	*p* value
VP (mmHg)				
Upstream stenosis	31.9 ± 10.9	19.5 ± 10.0	−39	0.019
Downstream stenosis	14.9 ± 8.6	14.3 ± 9.5	−4	0.705
A1 (au)				
Upstream stenosis	1.06 ± 1.37	0.18 ± 0.15	−83	0.003
Downstream stenosis	0.25 ± 0.17	0.13 ± 0.08	−48	0.096
A2 (au)				
Upstream stenosis	0.30 ± 0.50	0.05 ± 0.03	−83	0.14
Downstream stenosis	0.10 ± 0.09	0.05 ± 0.02	−48	0.15
Resp (au)				
Upstream stenosis	0.89 ± 0.60	0.57 ± 0.32	−36	0.226
Downstream stenosis	0.59 ± 0.46	0.54 ± 0.37	−9	1

**Conclusions:** Transverse sinus stenosis stenting reduces mean venous pressure and venous pressure pulsatility. This might have indirect effect on CSF dynamics and ICP amplitude. Sinus stenting yields complex venous pressure profile changes and possibly in brain biomechanics.

## A59 Influence of diabetes mellitus on CSF and brain characteristics

### E. A. Schmidt^1^, Z. Czosnyka^2^, L. Balardy^3^

#### ^1^Department of Neurosurgery, University Hospital Toulouse, France; ^2^Brain Physics Lab, Academic Neurosurgery, University of Cambridge, UK; ^3^Department of Geriatry, University Hospital Toulouse, France

##### **Correspondence:** E. A. Schmidt

*Fluids and Barriers of the CNS* 2017, 14(Suppl 1):A59

**Introduction:** Diabetes mellitus (DM) represents a major cause of morbidity and mortality worldwide. DM is an independent risk factor for cerebrovascular disease. DM is associated with an increase in the risk of brain atrophy and dementia. The WHO diagnostic criteria for DM is fasting plasma glucose ≥7.0 mmol/l. As well, glycated haemoglobin (HbA1c) reflects average plasma glucose over the previous 2–3 months. An HbA1c of 6.5% is recommended as the cut off point for diagnosing diabetes. DM is considered as a co morbidity of normal pressure hydrocephalus (NPH). We hypothesize that DM has an influence on ICP, CSF circulation and brain biophysical characteristics.

**Methods:** In a prospective cohort of 91 patients suspected of NPH, we performed lumbar infusion tests and measured at the same time plasma glucose and HbA1C. To understand the influence of DM on ICP and CSF/brain characteristics we dichotomized our cohort into two sub-groups: one diabetic and one non-diabetic. According the WHO, we characterized our two sub-groups with glycemia and HbA1C: patients with normal glycemia (i.e. <7.0 mmol/l) or high glycemia (i.e. ≥7.0 mmol/l) and patients with normal HbA1c (i.e. <6.5%) or high HbA1c (i.e. ≥6.5%). Then we compared the data in the diabetic and non-diabetic subgroups with Student test.

**Results:** The figure below gives mean ± SD values of various parameters in each diabetic and non-diabetic subgroup (Table 2).

**Table 2:** Intracranial pressure, CSF and brain characteristics in NPH patients with normal and high glycemia

	Glycemia < 7 mmol/l	Glycemia ≥ 7 mmol/l	*p* value	HbA1C < 6.5%	HbA1C ≥ 6.5%	*p* value
n	77	16		67	26	
ICP baseline (mmHg)	10.59 ± 3.49	10.31 ± 4.07	0.387	10.46 ± 3.32	10.77 ± 4.20	0.354
ICP plateau (mmHg)	29.10 ± 7.69	30.09 ± 8.75	0.325	28.76 ± 7.93	30.58 ± 7.59	0.159
AMP baseline (mmHg)	0.98 ± 0.52	0.85 ± 0.31	0.183	0.96 ± 0.51	0.93 ± 0.45	0.399
AMP plateau (mmHg)	4.10 ± 1.70	4.28 ± 1.94	0.358	3.99 ± 1.56	4.50 ± 2.12	0.102
Rcsf (mmHg*min/ml)	17.43 ± 11.94	20.35 ± 14.83	0.198	17.28 ± 12.29	19.60 ± 12.91	0.211
Elastance (1/ml)	0.22 ± 0.15	0.29 ± 0.24	0.069	0.20 ± 0.14	0.29 ± 0.24	0.015
PVI (ml)	15.02 ± 7.22	13.85 ± 8.25	0.283	15.25 ± 7.01	13.69 ± 8.27	0.181
Pss (mmHg)	1.78 ± 8.18	4.48 ± 5.65	0.106	1.29 ± 8.27	4.71 ± 6.11	0.029
CSF production (ml/min)	0.66 ± 0.68	0.30 ± 0.27	0.019	0.68 ± 0.69	0.37 ± 0.41	0.017

**Conclusions:** Our data suggest that patients with DM have stiffer brain with less CSF production rate and more resistive CSF outflow.

## A60 Subcommissural organ involvement in Niemann-pick type C disease

### D. Solomon^1^, L. Gray^2^, J. A. Buttner-Ennever^3^

#### ^1^Neurology, Johns Hopkins Hospital, Baltimore, MD, USA; ^2^Department of Physiology, Johns Hopkins University, School of Medicine, Baltimore, MD, USA; ^3^Anatomy and Cell Biology, Ludwig-Maximilians-University, Munich, Germany

##### **Correspondence:** D. Solomon

*Fluids and Barriers of the CNS* 2017, 14(Suppl 1):A60

**Introduction:** The subcommissural organ has been implicated in the development of congenital hydrocephalus, and some genetic models of hydrocephalus involve disrupting the development or function of this structure located along the ependyma of the caudal third ventricular at the posterior commissure. Ocular motor pathways important in vertical gaze traverse the posterior commissure; deficits in downward saccadic eye movement is a hallmark of Niemann-Pick Type C (NPC), an autosomal recessive neurodegenerative condition that interferes with trafficking and transport of cholesterol and glycolipids.

**Methods:** EM first manifest ataxia in her teens, with progressive neurologic symptoms of dysarthria, psychosis, inability to voluntarily initiate downward eye movements and hepatosplenomegaly.

**Results:** MRI brain showed atrophy, low signal abnormality in the thalamus, globus pallidus, putamen and external capsule bilaterally. Post-mortem examination showed glial plaque displacing the posterior commissure anteriorly, originating in the subcommissural organ (SCO, also called the subnucleus dorsalis or nucleus subcommissuralis of Carpenter). This structure is composed mainly of glia with fenestrated capillaries abutting the ventricle.

**Conclusions:** Alterations in secretory function, blood brain barrier and or glycoproteins (Reisner’s fiber) may contribute to hydrocephalus during development and metabolic neurodegeneration in adulthood.

## A61 Anthropomorphic in vitro survival study of shunt valves with and without anti siphoning device using post subarachnoid hemorrhage CSF

### D. Solomon^2^, M. G. Luciano^1^

#### ^1^Department of Neurosurgery, Johns Hopkins Hospital, Baltimore, MD, USA; ^2^Department of Neurology, Johns Hopkins Bayview Medical Center, Baltimore, MD, USA

##### **Correspondence:** D. Solomon

*Fluids and Barriers of the CNS* 2017, 14(Suppl 1):A61

**Introduction:** Devices have been developed to limit drainage of CSF in the upright posture using gravitational, membrane controlled and flow limiting designs. We chose to study the Codman SiphonGuard flow-regulated anti-siphoning device, to determine if the extra fluid channel makes it susceptible to blockage from elevated CSF protein.

**Methods:** Human CSF is collected and stored from patients in Johns Hopkins Neuroscience Critical Care Unit with an IVC following intraventricular or subarachnoid hemorrhage. Red blood cell count is reduced <1000 cc by freezing and centrifugation. Pooled CSF preserved with sodium azide (0.08%) is analyzed daily for desired protein concentration (5 g/l) and adjusted using sediment or dilution. Fluid, intraventricular catheters and shunts are maintained at body temperature (37 °C) in a shaking, thermal controlled water bath. Collection chamber height re: shunt valve is changed to mimic daily upright and recumbent postures. Gravitational flow is used, and physiological pulsatility mimicked by shaking water bath oscillations at 1 Hz. Testing lasts 30 days, after which valves will be pressure-flow tested per ISO-7197.

**Results:** Initial flow studies are currently underway; results will be available at the time of presentation.

**Conclusions:** We present an in vitro method for testing shunt survival in patients requiring CSF diversion after subarachnoid or intraventricular hemorrhage, taking into account effects of abnormal CSF contents, orthostatic postural changes and concomitant use of anti-siphoning devices with programmable shunt valves. Findings should help with the selection and timing of shunt systems in survivors of intracranial hemorrhage.

## A62 Two-year outcome of VA shunt for iNPH evaluated by modified Rankin scale

### K. Takagi^1^, K. Onouchi^2^

#### ^1^Normal Pressure Hydrocephalus Center, Kashiwa-Tanaka Hospital, Kashiwa, Japan; ^2^Department of Neurology, Kashiwa-Tanaka Hospital, Kashiwa, Japan

##### **Correspondence:** K. Takagi

*Fluids and Barriers of the CNS* 2017, 14(Suppl 1):A62

**Introduction:** Shunt surgery is the only treatment for iNPH. Although VA shunt has been almost abandoned, it has many advantages. We have selected VA shunt as a first choice surgery for iNPH. The purpose of this study is to report the 2-year outcome of VA shunt evaluate by mRS.

**Methods:** Between January 2012 to December 2013, 137 VA shunts were performed for NPH. From these patients, those less than 60 year, with secondary NPH, and with advanced Alzheimer’s disease were excluded. Patients with Evans Index less than 0.3 were included. Eighty-nine cases were the candidates for this study. The mRS was evaluated before the surgery (pre), 6 months (6 m), 1 year (1 y), and 2 years (2 y) after the surgery. The data was shown in mean (SD). The outcome at each time point was compared with preoperative condition. Statistical analysis was carried out with Dunnett’s multiple comparison test. Statistic significant level was set at p < 0.05.

**Results:** Mean age at the surgery was 78.08 (5.38) years old (male:female = 43:28). Mean mRSs for pre, 6 m, 1 year, and 2 years were 2.68 (0.99), 1.72 (1.19) (p < 0.0001), 1.71 (1.19) (p < 0.0001), and 1.90 (1.20) (p < 0.0001) respectively.

**Conclusions:** Although the patients were old at the surgery and those with Evans Index less than 0.3 were include, the VA shunt significantly improved the mRS of iNPH patients up to 2 years after surgery. This study suggests that VA shunt should be the first line for iNPH.

## A63 Telemetric monitoring of ICP within a shunt system. A single centre experience including the first in vivo comparison versus conventional intraparenchymal monitoring

### S. D. Thompson, L. D. Thorne, A. K. Toma, L. D. Watkins

#### The National Hospital for Neurology and Neurosurgery, Queen Square, London, UK

##### **Correspondence:** S. D. Thompson

*Fluids and Barriers of the CNS* 2017, 14(Suppl 1):A63

**Introduction:** Recent technological advances have allowed for telemetric, non-invasive monitoring of ICP. This allows clinicians to measure ICP non-invasively, once implanted, and assess for possible shunt dysfunction. Most ICP monitors show some drift in the accuracy in readings over time, however both the Spiegelberg intraparenchymal ICP bolt system and Miethke Sensor reservoir systems are reported to have zero and low drift respectfully.

**Methods:** The Miethke Sensor Reservoir^®^ allows telemetric ICP reading once implanted as part of a shunt system. A retrospective case series of a single unit’s experience with the Miethke Sensor Reservoir^®^ over a 4 year period was undertaken. This included the world’s first in vivo comparison between Sensor Reservoir^®^ and Spiegelberg intraparenchymal bolt.

**Results:** 13 Sensor Reservoirs^®^ have been inserted between February 2012 and June 2016, mean follow-up 14.5 months. There have been no reported complications.

One patient has undergone measuring of ICP concurrently using Sensor Reservoir^®^ and via a Spiegelberg intraparenchymal bolt 2.5 years after Sensor Reservoir^®^ implantation. Miethke Sensor Reservoir^®^ was within 1 mmHg when compared against the Spiegelberg bolt.

**Conclusions:** The Miethke Sensor Reservoir^®^ has been a reliable clinical tool in the measurement of ICP 2.5 years after implantation. Patients have had their symptoms managed in accordance with ICP, resulting in improved outcomes and less surgical interventions.

## A64 Creating an NPH pathway for new referrals

### S. D. Thompson, L. D. Thorne, L. D. Watkins, A. K. Toma

#### The National Hospital for Neurology and Neurosurgery, Queen Square, London, UK

##### **Correspondence:** S. D. Thompson

*Fluids and Barriers of the CNS* 2017, 14(Suppl 1):A64

**Introduction:** The NPH population is elderly, commonly suffering from multi-morbidities and are frail. Current practice at our hospital involves several out-patient appointments pre surgical admission, including initial surgeon review, anaesthetic assessment and baseline neuropsychological testing. We have commenced a one-stop polyclinic where patients can see all specialties in 1 day as well as being reviewed by an occupational therapist.

**Methods:** iNPH patients traveling from long distances to central London has proved difficult for some of these elderly patients. Therefore we have developed a one-stop NPH polyclinic, allowing patients to see neurosurgeons, specialist nursing, occupational therapy, anaesthetics and neuropsychology in 1 day. Here we undertake a prospective cohort observational study to review its impact.

**Results:** In the trial group, patients who attended the clinic were discharged, on average, day 4. This is compared against an average of day 5.6 for patients that did not pass through the clinic over the same period.

**Conclusions:** The one-stop clinic allowed for patients to be screened appropriately prior to admission. The input from occupational therapists assessing patients and putting adequate resources in place where needed pre admission, proved essential in reducing the length of admission for these patients.

## A65 Anatomic factors associated with post-hemorrhagic hydrocephalus among premature infants with intraventricular hemorrhage

### H. M. Tully^1^, T. L. Wenger^2^, W. A. Kukull^3^, D. Doherty^2^, W. B. Dobyns^2^

#### ^1^Department of Neurology, University of Washington, Seattle, WA, USA; ^2^Department of Pediatrics, University of Washington, Seattle, WA, USA; ^3^Department of Epidemiology, University of Washington, Seattle, WA, USA

##### **Correspondence:** H. M. Tully

*Fluids and Barriers of the CNS* 2017, 14(Suppl 1):A65

**Introduction:** Intraventricular hemorrhage (IVH) is a complication of prematurity often associated with ventricular dilatation, which may resolve over time or progress to post-hemorrhagic hydrocephalus (PHH). We investigated anatomic factors that could predispose infants with IVH to PHH.

**Methods:** We analyzed a cohort of premature infants diagnosed with Grade III-IV IVH between 2004 and 2014. Using ultrasounds and MRIs, we determined CSF obstruction pattern, skull shape and brain:skull ratios, comparing children with PHH to those with resolved ventricular dilatation (RVD), and comparing both groups to a set of healthy controls.

**Results:** Among 110 premature infants with grade III–IV IVH, 65 (59%) developed PHH. Infants with PHH had more severe ventricular dilatation compared to those with RVD, though ranges overlapped. Intraventricular CSF obstruction was observed in 36/42 infants (86%) with PHH and 0/18 (0%) with RVD (p < 0.001). The distribution of skull shapes found among infants with PHH was similar to that of infants with RVD, though markedly different from controls. No significant differences in supratentorial brain:skull ratio were observed; however, the mean infratentorial brain:skull ratio of infants with PHH was 5% greater (more crowded) than controls (p = 0.006), whereas the mean infratentorial brain:skull ratio of infants with RVD was 8% smaller (less crowded) than controls (p = 0.004).

**Conclusions:** Among premature infants with IVH, intraventricular obstruction and infratentorial crowding are strongly associated with PHH. Prospective studies are required to determine how these anatomic factors relate to the disease process and whether they can be used to predict the need for surgical intervention.

## A66 Ventricular-atrial shunting as primary option for the treatment of normal pressure hydrocephalus

### T. Vivas-Buitrago^1^, J. Lu^1^, J. Robison^1^, A. Hung^1^, A. Adam^2^, E. Sankey^1^, D. Moran^1^, S. Vakili^1^, M. A. Patel^1^, I. Jusué-Torres^1^, B. Elder^1^, C. R. Goodwin^1^, D. Rigamonti^1,3^

#### ^1^Department of Neurosurgery, Johns Hopkins University, School of Medicine, Baltimore, MD, USA; ^2^Johns Hopkins Biostatistics Center, Johns Hopkins Bloomberg School of Public Health, Baltimore, MD, USA; ^3^Johns Hopkins Aramco Healthcare, Dhahran, Saudi Arabia

##### **Correspondence:** T. Vivas-Buitrago

*Fluids and Barriers of the CNS* 2017, 14(Suppl 1):A66

**Introduction:** Ventricular-peritoneal (VP) and ventricular-atrial (VA) shunting are the primary procedures for NPH. Given the invasiveness of the VA placement into the cardiovascular system and its possible complications, most surgeons have reserved this technique for cases with history of VP failure, intra-abdominal scaring/infection, and obese patients. This study aims to compare the complication rates between these two techniques.

**Methods:** A retrospective chart review was conducted on all patients undergoing primary shunt-placement treatment for NPH by the senior author between 1993 and 2014. Analysis includes any complication related to the procedure.

**Results:** 150 patients underwent VA shunt-surgery, while 435 patients underwent VP. Age ranged from 48 to 87 years for VA and 35 to 87 years for VP with an age-average of 74 and 70 years respectively. 26% of VA patients had post-surgical complications vs. a 25% of VP population. Subdural-Hematoma (SDH) was most common VA complication (17%) without need of surgical-evacuation vs. 7% of SDH in VP shunt with surgical-evacuation needed in 19% of these cases. Shunt-obstruction: (VA: 7%) vs. (VP: 19%). Overdrainage: (VA: 9%) vs. (VP: 7%). CSF infection (VA: 1%) vs. (VP: 5%). Malpositioned-catheter: (VA: 0.71% vs. VP: 1.31%). Shunt surgical revisions were needed in 11% of VAs vs. 29% for VPs.

**Conclusions:** Our study suggests that VA compares favorably to VP shunting in regards to postsurgical complications. Furthermore, no cardiopulmonary complications related to VA placement were found. Given these facts, in our experience, we can conclude that in the absence of contraindications, ventricular-atrial shunting should be consider as the primary option for the treatment of NPH.

## A67 Ventricular volumetric analysis during hydrocephalus development in an animal model of adult chronic hydrocephalus

### T. Vivas-Buitrago^1^, I. Jusué-Torres^1^, J. Lu^1^, J. Robison^1^, A. Adam^8^, J. A. Crawford^3^, M. V. Pletnikov^3^, J. Xu^4^, A. Blitz^5^, D. A. Herzka^6^, H. Guerrero-Cazares^1^, A. Quiñones-Hinojosa^1,7^, S. Mori^2^, D. Rigamonti^1,9^

#### ^1^Department of Neurosurgery, Johns Hopkins University, School of Medicine, Baltimore, MD, USA; ^2^Department of Radiology-Magnetic Resonance Research, Johns Hopkins University, School of Medicine, Baltimore, MD, USA; ^3^Department of Psychiatry and Behavioral Sciences, Johns Hopkins University, School of Medicine, Baltimore, MD, USA; ^4^F. M. Kirby Research Center for Functional Brain Imaging at the Kennedy Krieger Institute, Johns Hopkins University, School of Medicine, Baltimore, MD, USA; ^5^Department of Radiology and Radiological Science, Johns Hopkins University, School of Medicine, Baltimore, MD, USA; ^6^Department of Biomedical Engineering, Johns Hopkins University, Baltimore, MD, USA; ^7^Department of Oncology, Johns Hopkins University, School of Medicine, Baltimore, MD, USA; ^8^Johns Hopkins Bloomberg School of Public Health, Baltimore, MD, USA; ^9^ Johns Hopkins Hopkins Aramco Healthcare, Dhahran, Saudi Arabia

##### **Correspondence:** T. Vivas-Buitrago

*Fluids and Barriers of the CNS* 2017, 14(Suppl 1):A67

**Introduction:** The pathogenesis of ventricular enlargement and its correlation to symptomatology is not entirely understood. We correlated the temporal enlargement of the ventricular system (VS) with symptomatology.

**Methods:** Kaolin was injected bilaterally into the subarachnoid space overlying the cranial convexities in 18 adult rats. MRI was performed on an 11.7-T scanner at 14, 60, 90 and 120 days post-injection. Volumes of the VS were measured and correlated with behavioral results biweekly.

**Results:** Right and left ventricles showed non-linear trajectories, with the highest increase at 60 days, (p ≤ 0.001, and, p = 0.001, respectively). 3rd ventricle had a linear change volume, (p = 0.002), synchronous with lateral ventricular enlargement. No significant change occurred in the 4th ventricle (p = 0.086). No symptoms occurred in the first 68 days. Median time to first symptom: 92 days. Most common symptom was anxiety = (83.3%), while the most common first symptom was gait change = (50%). There was a significant increase in odds for all clinical symptoms over time (p < 0.001). Significant relationship was only observed between MRI volume and locomotor symptoms at any time point during the follow-up. Additionally, the odds of locomotor symptoms increase by 3% for every 1 additional mm^3^ of volume in left ventricle, (p = < 0.001). Similar estimate for the right ventricle at 1% increase per 1 mm^3^, (p = 0.023) and slightly lower for the total MRI volume at 2%, (p = 0.013).

**Conclusions:** Periodic ventricular volumetric measurement is feasible in this model given the slow progressive development of hydrocephalus. Late symptomatology presentation in this model allows a continuous in vivo monitoring of the dynamics between ventricular enlargement and symptomatology.

## A68 Sub-ventricular zone (SVZ) stem cell exploration in a novel animal model of adult chronic communicating hydrocephalus

### T. Vivas-Buitrago^1^, I. Jusué-Torres^1^, J. Lu^1^, J. Robison^1^, A. Adam^7^, J. A. Crawford^3^, M. V. Pletnikov^3^, P. Saavedra^1^, H. Treviño^1^, K. Maitani^1,8^, J. Xu^4^, A. Quiñones-Hinojosa^1,6^, H. Guerrero-Cazares^1^, D. Rigamonti^1,2^

#### ^1^Department of Neurosurgery, Johns Hopkins University, School of Medicine, Baltimore, MD, USA; ^2^Johns Hopkins Aramco Healthcare, Dhahran, Saudi Arabia; ^3^Department of Psychiatry and Behavioral Sciences, Johns Hopkins University, School of Medicine, Baltimore, MD, USA; ^4^F. M. Kirby Research Center for Functional Brain Imaging at the Kennedy Krieger Institute, Johns Hopkins University, School of Medicine, Baltimore, MD, USA; ^5^Department of Radiology and Radiological Science, Johns Hopkins University, School of Medicine, Baltimore, MD, USA; ^6^Department of Oncology, Johns Hopkins University, School of Medicine, Baltimore, MD, USA;^7^Johns Hopkins Biostatistics Center, Johns Hopkins Bloomberg School of Public Health, Baltimore, MD, USA; ^8^Tohoku University School of Medicine, Sendai, Japan

##### **Correspondence:** T. Vivas-Buitrago

*Fluids and Barriers of the CNS* 2017, 14(Suppl 1):A68

**Introduction:** Normal pressure hydrocephalus (NPH) is a communicating form of hydrocephalus with slow-onset ventriculomegaly and late symptomatology. The subventricular zone (SVZ) of the lateral ventricles has been established as the primary site for adult neurogenesis. Few studies have focused on the potential impact on the SVZ throughout the course of NPH. We studied the SVZ stem cell population in a novel animal model of adult chronic communicating hydrocephalus.

**Methods:** We injected Kaolin bilaterally into the subarachnoid space overlying the cranial convexities in 15 adult rats. We performed sham surgery (saline_injection) in five animals, and used five additional not-injected animals as controls. We obtained MRI using a 11.7T scanner at 15, 60, 90 and 120 days post kaolin injection (KI). Behavioral evaluations were performed independently every 2 weeks. KI animals were sacrificed at 15, 90 and 120 days after the injection. Immunofluorescence staining against neural progenitor markers, Nestin and GFAP, as well as proliferation markers, Ki67, was utilized to identify proliferating SVZ cells.

**Results:** 86% of the KI animals demonstrated radiological evidence of ventricular enlargement (VE). Evans index showed a linear VE progression over time. Initial clinical presentation was locomotor activity decline at 1 month, followed by balance, coordination, and memory deficits at 3 months. SVZ stem cell proliferation rate remained similar between experimental and control groups throughout the study.

**Conclusions:** Chronic communicating hydrocephalus in our model eventually leads to locomotor and cognitive changes without affecting the proliferation rate of the SVZ stem cells.

## A69 Ventricular-atrial shunting complications rate in normal pressure hydrocephalus. A single surgeon practice experience

### T. Vivas-Buitrago^1^, J. Lu^1^, J. Robison^1^, A. Hung^1^, A. Adam^2^, E. Sankey^1^, D. Moran^1^, S. Vakili^1^, M. A. Patel^1^, I. Jusué-Torres^1^, B. Elder^1^, C. R. Goodwin^1^, D. Rigamonti^1,3^

#### ^1^Department of Neurosurgery, Johns Hopkins University, School of Medicine, Baltimore, MD, USA; ^2^Johns Hopkins Biostatistics Center, Johns Hopkins Bloomberg School of Public Health, Baltimore, MD, USA; ^3^Johns Hopkins Aramco Healthcare, Dhahran, Saudi Arabia

##### **Correspondence:** T. Vivas-Buitrago

*Fluids and Barriers of the CNS* 2017, 14(Suppl 1):A69

**Introduction:** Cerebrospinal fluid diversion has been the gold-standard treatment for normal pressure hydrocephalus (NPH). Ventriculoatrial shunting (VAS) is one of these diversion strategies. VAS is thought to be an invasive procedure associated with high risk of severe cardiopulmonary complications. However, few studies have focused on this topic, being the majority out of date considering the recent surgical and technological advances. Further research is required to better understand the current rate of complications associated to the VAS.

**Methods:** A retrospective chart review was conducted on all patients undergoing a VA shunt placement as primary treatment for NPH by the senior author between 1993 and 2014 at the Johns Hopkins Medical Institution. Analysis includes any complication related to the procedure within the period immediately after surgery until last follow-up.

**Results:** 150 patients underwent VA shunt-surgery. 87 were male. Age ranged from 48 to 87 years. 26% of the patients had post-surgical complications. Most common complication was subdural-hematoma/hygroma (SDH) presented in 17% of all VAS patients, with no need of surgical evacuation. Shunt-obstruction occurred in 7% of patients, over-drainage in 9%. Surgical revisions were needed in 11% of all cases.

**Conclusions:** No cardio-pulmonary complications occurred in this study. Further analysis and comparison with our ventriculoperitoneal shunted patient’s data will be done to compare the complication rates between these two techniques.

## A70 Evaluation of hydrocephalus after severe obstructive intraventricular hemorrhage as a risk factor for poor functional outcome in the clear III trial

### W. C. Ziai^1^, V. Eslami^1^, S. Nekoovaght-Tak^1^, R. Dlugash^1^, G. Yenokyan^2^, N. McBee^1^, D. F. Hanley^1^

#### ^1^Department of Neurology, The Johns Hopkins University School of Medicine, Baltimore, MD, USA; ^2^Department of Biostatistics, The Johns Hopkins University Bloomberg School of Public Health, Baltimore, MD, USA

##### **Correspondence:** W. C. Ziai

*Fluids and Barriers of the CNS* 2017, 14(Suppl 1):A70

**Introduction:** The impact of acute hydrocephalus on outcomes in adult spontaneous intraventricular hemorrhage is unstudied. We explore association between imaging biomarkers of hydrocephalus and functional outcomes in the Clot Lysis: Evaluating Accelerated Resolution of Intraventricular Hemorrhage (CLEAR) III trial.

**Methods:** We analyzed diagnostic and day 30 CT scans from all 500 patients. We measured bicaudate index, fronto occipital horn ratio, and Evan’s index and estimated association of indices with blinded assessment of stroke severity at day 30 and 180. Generalized linear models were used to estimate whether Evan’s index correlates with functional outcomes, after adjusting for confounders.

**Results:** Evan’s index at day 30 had stronger correlation with both diagnostic and end of treatment IVH volume than other indices. Only bicaudate index was significantly associated with alteplase treatment (median[iqr] 0.21[.18, .24] vs. 0.22[.19, .25]; p = 0.02). Indices did not differ by permanent shunt status. Patients were categorized into tertiles of Evans index. Proportion of patients with poor day 30 modified Rankin Scale increased in successive tertiles (p < 0.001). Adjusted odds ratio for highest tertile vs. lowest was 3.19 (95% CI 1.45, 7.05; p = 0.004). Day 30 Barthel Index, Mini mental status exam, Stroke Impact Scale (SIS) and all but one domain-specific SIS score were inversely associated with Evan’s and bicaudate index. Day 180 functional outcomes were not associated with hydrocephalus indices.

**Conclusions:** Extent of ventricular dilatation after IVH is a strong risk factor for poor early functional outcome independent of known severity factors. Persistent hydrocephalus may adversely affect outcomes despite early use of EVD, and intraventricular alteplase treatment.

